# Precision immune regulation in KRAS-mutated cancers: the final piece of the puzzle?

**DOI:** 10.1186/s13046-025-03444-1

**Published:** 2025-07-03

**Authors:** Shenao Fu, Jiayao Ma, Changjing Cai, Jun Tan, Xiangying Deng, Hong Shen, Shan Zeng, Yihong Chen, Ying Han

**Affiliations:** 1 Department of Oncology, Xiangya Hospital, Central South University, Changsha, China; 2 National Clinical Research Center for Geriatric Disorders, Xiangya Hospital, Central South University, Changsha, China; 3 Department of Neurosurgery, Xiangya Hospital, Central South University, Changsha, China

**Keywords:** KRAS, Cancer, Targeted therapy, Immunotherapy, Microenvironment

## Abstract

In recent years, the development of targeted therapies for tumors with KRAS mutations has progressed rapidly, rendering the notion of KRAS as “undruggable” outdated. However, targeted therapies for KRAS mutations still face numerous challenges, including resistance, efficacy concerns, toxicity issues, and hurdles in drug development. Exploring alternative treatment modalities is thus essential. Extensive research has demonstrated that KRAS mutations significantly influence the immune microenvironment, presenting both challenges and opportunities for immunotherapy. Interestingly, it has been observed that different KRAS mutations and co-mutation subtypes exhibit significant variations in their immunological microenvironments, which undoubtedly impact immunotherapy choices. Here, we review the history of KRAS-targeted therapy, highlighting existing challenges, and summarize changes in the immune microenvironment of KRAS-mutated cancers and their potential therapeutic targets. We compare differences in the immune microenvironment across various mutation types and co-mutation subtypes, and offer perspectives on future research directions.

## Introduction

KRAS is one of the most classical, significant, and high-frequency oncogenes, causing about one million deaths annually worldwide [1]. Structurally, it has the second-highest mutation rate in human tumors, ranking first in pancreatic cancer and colon adenocarcinoma, after TP53, a major tumor suppressor gene. KRAS codon G12 is the most frequently mutated site in human tumors, accounting for approximately 90% of KRAS mutations and 12% of all cancer patients [2]. Functionally, wild-type KRAS binds GTP, activating downstream signaling pathways, while binding to GDP shuts down these signals. Because wild KRAS proteins have GTP-hydrolysis activity, activation of related pathways is temporary and controllable. Oncogenic mutations cripple KRAS GTPase activity, locking it in a GTP-bound state. This fuels the persistent activation of critical downstream pathways such as MAPK-ERK and PI3K/AKT, driving aggressive tumor phenotypes including proliferation, invasion, migration, and immune evasion [3–7]. KRAS mutants were once described as “untargetable” targets due to the difficulty in finding small molecules that bind effectively to mutated KRAS [4, 8]. After decades of targeted drug development, progress has been made in targeting KRAS^G12C^ and KRAS^G12D^[9]. And multi-KRAS inhibitor RMC-7977 exhibited exciting preclinical results [10, 11]. Despite these advances, the success of KRAS-targeted therapies remains uncertain, as the inhibitors developed for KRAS^G12C^ and KRAS^G12D^ do not directly apply to other mutations, and drug resistance remains a challenge [12–14].

Unlike the targeting idea that only focuses on tumor cells, in recent years, with the progress of technology and the continuous improvement of tumor development theory, the impact of tumor immune microenvironment on tumors has been paid more and more attention [15]. KRAS mutations shape the immune microenvironment, affecting tumor progression and treatment efficacy [16, 17]. While not yet a definitive guide for first-line treatments, KRAS mutations in NSCLC show promising responsiveness to immune checkpoint inhibition (ICI) [18]. Moreover, combination therapies have shown enhanced efficacy in KRAS-mutant tumors [19]. Interestingly, the immune microenvironment shaped by different KRAS mutations varies, sometimes producing opposing effects [20]. Specifically, the two most frequent subtypes of mutations, KRAS^G12D^ and KRAS^G12C^ exhibit opposite PD-L1 expression patterns [21]along with consequent differences in response to ICI therapy [22]. Similarly, co-mutations such as TP53, LKB1, and KEAP1 further alter the immune landscape [23] influencing treatment outcomes [24].

Current evidence highlights the immune microenvironment as a key factor in overcoming the therapeutic challenges of KRAS-mutant cancers. Targeting the immune microenvironment in combination with existing treatments may lead to breakthroughs. Additionally, understanding the heterogeneity of the immune microenvironment in KRAS-mutant cancers presents challenges but offers opportunities for precision treatment. This review summarizes the immune microenvironment in KRAS-mutant cancers and the latest advances in immunotherapy, emphasizing subtype heterogeneity and potential future directions in treatment.

## Overview of KRAS mutant cancers and the development history of targeted therapies

### The role of the KRAS gene and its mutations in cancer

The RAS family members, KRAS, NRAS, and HRAS, comprise small GTPases that act as binary switches in cellular signaling [25]. When bound to GTP, RAS proteins enter the “on” mode, initiating downstream signal transmission; upon hydrolysis of GTP to GDP, they transition to “off” mode, halting further signal propagation. This signaling function, governed by specific switch domains I and II, is facilitated by protein-protein interactions [1].

**Fig. 1:**
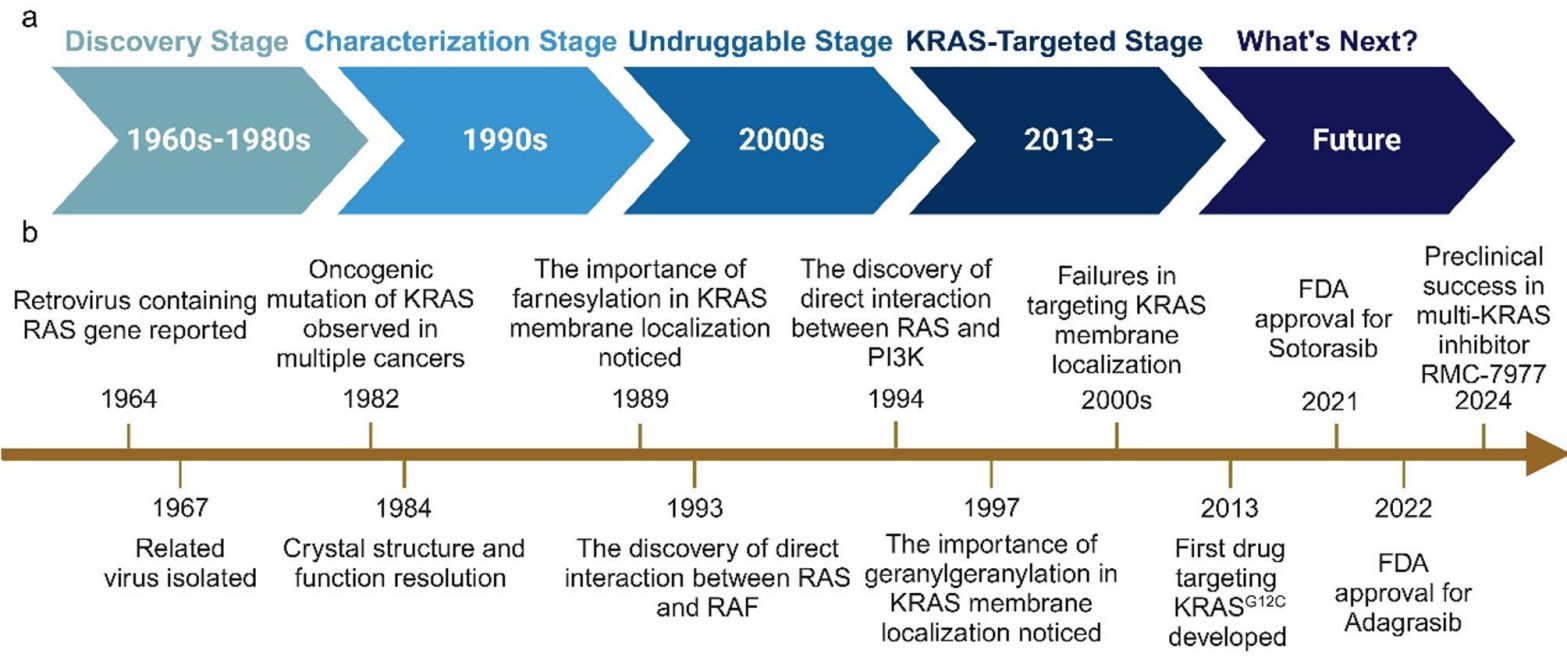
Timeline of research on KRAS mutations in cancer. Research on KRAS has been categorized into four developmental stages (**a**) based on the major advances in each period. We summarize the research content at each stage and highlight key breakthroughs (**b**)

RAS was initially identified for its oncogenic properties (Fig. 1), with KRAS mutations constituting the most prevalent, comprising 85% of all RAS oncogenic mutations and affecting 12% of all cancer patients [2, 26]. Codons 12, 13, 61, 117, and 146 emerge as the most frequent mutation sites, reflecting distinct preferences across tumor types [27]. For instance, pancreatic cancers frequently exhibit the KRAS^G12D^ mutation, whereas lung adenocarcinomas more commonly feature the KRAS^G12C^ mutation associated with smoking [28, 29]. This variability highlights the complexity of KRAS mutations and their differential impact on tumor biology.

Oncogenic KRAS mutations lead to structural changes that impair GTPase activity, keeping KRAS in a constantly active “on” state and triggering downstream signaling [3]. The extensive range of these signals means that mutant KRAS impacts a variety of malignant phenotypes in tumor cells. Apart from classical pathways related to MAPK and AKT that influence growth, KRAS mutations also regulate metabolic reprogramming [5], stemness [6], and immune evasion [7], making KRAS a key target for therapeutic intervention.

Due to the powerful regulatory role and high incidence, KRAS mutation status is a potential biomarker for patient stratification. Compared to KRAS wild-type, the effectiveness of chemotherapy in KRAS-mutant NSCLC was inferior [30]. However, the KEYNOTE-042 trial [31] and a retrospective study in China [32] demonstrated that immunotherapy offered better outcomes for KRAS-mutant NSCLC patients than chemotherapy. In combination therapies, chemotherapy combined with immunotherapy has shown advantages over chemotherapy monotherapy or combined with anti-angiogenesis [33]. An increasing body of research suggested that immunotherapy could be a viable treatment option for KRAS-mutant NSCLC patients. Traditional chemotherapy could be used for KRAS-mutant colorectal cancer; however, it was less effective than in KRAS wild-type cases [34]. Ongoing clinical trials are evaluating the efficacy of combinations, like sintilimab plus bevacizumab and CapeOx [35] or durvalumab and tremelimumab with chemotherapy [36] as first-line treatments for metastatic colorectal cancer with RAS mutations and microsatellite stable (MSS) states. Early phase trials suggested that combined immunotherapy might outperform chemotherapy [36]. However, due to the lack of a control group, the final comparison of efficacy awaits further trial results.

### Origins of KRAS subtype differences: why is precise subtyping essential?

This review highlights the heterogeneity of the immune microenvironment among various subtypes of KRAS mutations and co-mutations, emphasizing the role of KRAS in driving this variability. While the contribution of co-mutations as a source of heterogeneity is well-recognized [23], the heterogeneity associated with different types of KRAS mutations warrants further clarification. Despite being uniformly categorized as activating mutations, both oncogenic KRAS mutations and simple increases in KRAS copy number may result in divergent and sometimes opposing biological outcomes. This section summarizes the origins of these differences and explores mechanisms driving KRAS mutation subtypes.

The heterogeneity of immune microenvironment stems from upstream signaling pathways, particularly KRAS pathway activation. KRAS’s conformation differs between its GDP-bound and GTP-bound states, regulated by guanine nucleotide exchange factors (GEFs) and GTPase-activating proteins (GAPs) [37]. Among the most well-characterized GEFs, SOS1 promotes KRAS activation by facilitating its transition to the GTP-bound state. This crucial mechanism explains the historical focus on SOS1 as a therapeutic target, particularly before KRAS itself was recognized as druggable [38–40]. Conversely, GAPs, such as NF1, counteract GEF activity by enhancing the intrinsic GTPase activity of KRAS, thereby accelerating the hydrolysis of GTP to GDP and cycling KRAS back to its inactive state. The loss of GAP function leads to increased KRAS activity and tumorigenesis [41]. Notably, in KRAS oncogenic mutations, the loss of intrinsic GTPase activity is not solely due to the mutation. Amino acid substitutions lead to structural alterations that introduce spatial hindrance, disrupting the interaction between GAPs and KRAS and thereby impairing GTPase activity. The magnitude of this spatial hindrance varies across different KRAS mutation subtypes, resulting in differential activation levels of KRAS. This variation in activation levels across KRAS mutation subtypes contributes to differing biological outcomes and highlights the need for precise subtyping in KRAS-driven cancers [42].

KRAS mutations also affect its interaction with downstream signaling molecules, determining the selection of downstream pathways activated by oncogenic KRAS signaling. Structural studies of KRAS have revealed that its interaction regions with key downstream effectors, such as PI3K and RAF, are primarily located near the Switch I and Switch II regions [43, 44]. Importantly, the most frequently mutated residues in KRAS-driven cancers—codons 12, 13, and 61—are situated within or adjacent to these regions. For instance, mutations at these residues alter the affinity of KRAS for RAF, a pivotal effector in the MAPK signaling pathway. These alterations in affinity are not uniform across mutation subtypes. Mutations such as G12A, G12C, G13D, and Q61L exhibit relatively minor reductions in RAF affinity, maintaining a high-RAF-affinity state similar to that of wild-type KRAS. In contrast, mutations such as G12R, G12V, and G12D display markedly reduced RAF affinity. These differences in affinity directly influence the “preference” of KRAS mutation subtypes for engaging downstream effectors, thereby driving distinct oncogenic signaling outcomes [42]. These differences in pathway activation emphasize the importance of precise KRAS subtyping for both the biology of KRAS-driven cancers and the development of targeted therapeutic strategies.

In summary, the heterogeneity among KRAS mutation subtypes arises from two fundamental differences: the variations in intrinsic enzymatic activity and regulatory protein interactions, which determine the degree of KRAS pathway activation; and the differences in binding affinities to downstream effectors, which dictate the preference for activating specific downstream pathways. Conventional paradigms of oncogenic KRAS mutations often emphasize their tumorigenic potential, neglecting the distinct biological consequences across mutation subtypes. However, these upstream differences can lead to profoundly divergent, even opposing, immune microenvironmental phenotypes in KRAS-mutant tumors, a topic that will be elaborated in subsequent sections.

### A brief history of targeted therapy development for KRAS mutations

Despite high expectations, KRAS-targeted therapy development has faced significant challenges (Fig. 1). Mutant KRAS protein has been deemed “undruggable” owing to its small size and smooth surface, which lacks deep pockets for effective drug binding [8]. This limitation spurred interest in downstream inhibitors of KRAS, such as RAF, MEK, and ERK inhibitors, despite achieving modest success [45, 46]. For instance, combining selumetinib, a MEK inhibitor, with docetaxel did not enhance overall survival in previously treated KRAS mutant non-small-cell lung cancer (NSCLC) patients [46]. Moreover, resistance to monotherapy and the high toxicity of combined therapies emerged as new challenges [47–49].

A turning point occurred in 2013 when Kevan Shokat’s team identified a series of compounds targeting the small pocket formed by switch-II and Cys 12 in KRAS^G12C^ protein [50]. This groundbreaking discovery began a golden decade of KRAS-targeted research, during which several compounds featuring a similar acrylamide warhead were developed [51]. The newly developed compounds bound to KRAS^G12C^, but with unstable performance in vivo [52]. Based on compounds identified by Shokat and colleagues, research on modifications for improving the performance in vivo has succeeded. By 2018, ARS-853 and ARS-1620 demonstrated in vivo inhibition of KRAS^G12C^ [53, 54], followed by the successful clinical trial outcomes of MRTX849 (Adagrasib) [55] and AMG 510 (Sotorasib) [56, 57] which received FDA approval in 2022 and 2021, respectively, marking the targeting of previously undruggable KRAS. Furthermore, in 2021, MRTX1133 emerged as a novel noncovalent inhibitor targeting the common KRAS^G12D^ mutation [58]. The selectivity and efficacy have been demonstrated in vitro [59, 60] and in vivo [59, 61, 62], followed by phase I/II clinical trials initiated in 2023 (NCT05737706).

Ongoing development of novel KRAS-targeted drugs is encouraging (Tables 1 and 2). Innovatively, a small molecule capable of remodeling cyclophilin A (CYPA), a cellular chaperone, was designed to form a high-affinity, selective CYPA: drug: KRAS tricomplex [63] showing promising anti-tumor efficacy in cell and PDX models, and leading to ongoing phase 1 clinical trials (NCT05462717 and NCT05379985). In this mechanism, the drug acted as a small molecule linker to tether the intracellular protein CYPA with KRAS, thereby “locking” KRAS and preventing downstream signaling transduction [10]. This design paradigm offers a novel approach to the discovery of KRAS drugs. Recently, through structure-guided drug modifications, based on a previous series of drugs, a small-molecule drug RMC-7977 with the same mechanism of action has been designed to target various KRAS mutations and even wild-type KRAS [10]. Additionally, recent years have seen the advent of emerging pan-KRAS mutation inhibitors, such as BI-2865 [64], which hold substantial promise for treating various KRAS mutations.

Despite these advances, challenges persist in the ongoing assessment of multi- or pan-KRAS inhibitors. The wide-range targeting brought uncertainties related to toxicity arising from alterations in the extensive RAS signaling pathway [12–14]. While there is no bedside evidence proving the explicit toxicity of pan- or multi-KRAS inhibitors [11] direct targeting of the wild-type KRAS with powerful regulating function inevitably raises concerns. Furthermore, precise mutation-specific targeting of other mutations in KRAS seemed a distant prospect due to the unique nature of the small pocket formed with Cys12, rendering the success achieved in KRAS^G12C^ inhibitor development unattainable in other mutation types. Additionally, newly approved drugs also encountered resistance issues [65, 66] with a response rate of 50% in KRAS^G12C^ NSCLC patients [67], and the efficacy was even lower in CRC [57]. The efficacy of Sotorasib in the phase 3 trial compared with docetaxel for treating NSCLC showed modest advantages [68], far from solving the problems. The resistance mechanism to mutation-specific KRAS inhibitors was typically believed to stem from compensatory activation of the KRAS pathway [69, 70]. Due to selective pressure against a specific mutation type, subclones containing other types of KRAS mutations or mutations in other downstream pathway members might survive and become dominant clones [66, 71]. RMC-7977 was expected to address the resistance observed with previous mutation-specific KRAS inhibitors due to its targeting properties towards multi-KRAS and wild-type KRAS [10]. However, RMC-7977 also exhibited significant resistance. Interestingly, in this scenario, tumor cells selected as dominant clones showed activation of YAP/TAZ and Myc [11].

Future improvements in drug efficacy through optimization, combination, and identification of target populations are critical. In addition to continuing to address the challenges of targeting KRAS directly, exploring new therapeutic strategies is essential.

**Table 1:** Clinical trials of new drugs targeting mutant KRAS

Therapy	Mutant type	Tumor type	NCT number	Phase	Mechanism	Sponsor	Status	Reference*
MK-1084 combined with Pembrolizumab	KRAS^G12C^	NSCLC	NCT06345729	Phase 3	KRAS^G12C^ inhibitor combined with PD-1 monoclonal antibody	Merck Sharp & Dohme LLC	Not yet recruiting	[72]
D-1553	KRAS^G12C^	NSCLC	NCT06300177	Phase 3	KRAS^G12C^ inhibitor	Chia Tai Tianqing Pharmaceutical Group Co., Ltd.	Not yet recruiting	[73, 74]
JDQ443	KRAS^G12C^	NSCLC brain metastasis	NCT05999357	Phase 2	KRAS^G12C^ inhibitor	Maastricht University Medical Center	Not yet recruiting	[75]
JAB-21,822	KRAS^G12C^	Pancreatic cancer	NCT06008288	Phase 2	KRAS^G12C^ inhibitor	Jacobio Pharmaceuticals Co., Ltd.	Recruiting	[76]
ZG19018	KRAS^G12C^	Solid tumors	NCT06237400	Phase 1/2	KRAS^G12C^ inhibitor	Suzhou Zelgen Biopharmaceuticals Co., Ltd	Recruiting	[77]
D-1553 combination with IN10018	KRAS^G12C^	Solid tumors	NCT06166836	Phase 1/2	KRAS^G12C^ inhibitor combined with FAK inhibitor	InxMed (Shanghai) Co., Ltd.	Recruiting	[73, 74]
FMC-376	KRAS^G12C^	Solid tumors	NCT06244771	Phase 1/2	KRAS^G12C^ inhibitor	Frontier Medicines Corporation	Recruiting	[78]
RMC-6291 or RMC-6236 combined with Pembrolizumab, with or without chemotherapy	KRAS^G12C^	NSCLC	NCT06162221	Phase 1/2	KRAS^G12C^ inhibitor combined with PD-1 monoclonal antibody, with or without chemotherapy	Revolution Medicines, Inc.	Recruiting	[79, 80]
BBO-8520	KRAS^G12C^	NSCLC	NCT06343402	Phase 1	KRAS^G12C^ inhibitor	TheRas, Inc	Recruiting	[81]
BEBT-607	KRAS^G12C^	Solid Tumor	NCT06117371	Phase 1	KRAS^G12C^ inhibitor	BeBetter Med Inc	Recruiting	/
BI 1,823,911 monotherapy or combined with BI 1,701,963	KRAS^G12C^	Solid tumors	NCT04973163	Phase 1	KRAS^G12C^ inhibitor monotherapy or combined with SOS1 inhibitor	Boehringer Ingelheim	Active, not recruiting	[82]
GEC255	KRAS^G12C^	Solid tumors	NCT05768321	Phase 1	KRAS^G12C^ inhibitor	GenEros Biopharma Hangzhou Ltd	Recruiting	[83]
LY3537982	KRAS^G12C^	Solid tumors	NCT06235983	Phase 1	KRAS^G12C^ inhibitor	Eli Lilly and Company	Not yet recruiting	[84, 85]
RMC-6291 in combination with RMC-6236	KRAS^G12C^	Solid tumors	NCT06128551	Phase 1	KRAS^G12C^ inhibitor combined with pan-KRAS inhibitor	Revolution Medicines, Inc.	Recruiting	[79, 80]
RMC-9805	KRAS^G12C^	Solid tumors	NCT06040541	Phase 1	KRAS^G12C^ inhibitor	Revolution Medicines, Inc.	Recruiting	[86]
SY-5933	KRAS^G12C^	Solid tumors	NCT06006793	Phase 1	KRAS^G12C^ inhibitor	Shouyao Holdings (Beijing) Co. LTD	Recruiting	/
HYP-2090PTSA	KRAS^G12C^	Solid tumors	NCT06243354	Phase 1/2	KRAS^G12C^ and PI3K dual-site inhibitors	Sichuan Huiyu Pharmaceutical Co., Ltd	Recruiting	/
MRTX1133	KRAS^G12D^	Solid tumors	NCT05737706	Phase 1/2	KRAS^G12D^ inhibitor	Mirati Therapeutics Inc.	Recruiting	[58, 87]
HRS-4642	KRAS^G12D^	Solid tumors	NCT05533463	Phase 1	KRAS^G12D^ inhibitor	Jiangsu HengRui Medicine Co., Ltd.	Recruiting	[88]
INCB161734 monotherapy or combined with Cetuximab and Retifanlimab	KRAS^G12D^	Solid tumors	NCT06179160	Phase 1	KRAS^G12D^ inhibitor monotherapy or combined with EGFR monoclonal antibody and PD-1 monoclonal antibody	Incyte Corporation	Recruiting	[89]
QTX3034monotherapy or combined with Cetuximab	KRAS^G12D/(V)**^	Solid tumors	NCT06227377	Phase 1	KRAS^G12D^ inhibitor monotherapy or combined with EGFR monoclonal antibody	Quanta Therapeutics	Recruiting	[90]
BI 3,706,674	Pan-KRAS	Gastric, esophageal, and gastroesophageal junction adenocarcinoma	NCT06056024	Phase 1	Pan-KRAS inhibitor	Boehringer Ingelheim	Recruiting	[91]
YL-17,231	Pan-KRAS	Solid tumors harboring mutations in KRAS, HRAS, or NRAS	NCT06105022	Phase 1	Pan-KRAS inhibitor	Shanghai YingLi Pharmaceutical Co. Ltd.	Not yet recruiting	[92]
YL-17,231	Pan-KRAS	Solid tumors harboring KRAS mutations	NCT06078800	Phase 1	Pan-KRAS inhibitor	Shanghai YingLi Pharmaceutical Co. Ltd.	Recruiting	[92]
ZG2001 Tosilate Tablets	Pan-KRAS	Solid tumors	NCT06237413	Phase 1/2	SOS1 inhibitor	Suzhou Zelgen Biopharmaceuticals Co., Ltd	Recruiting	/
BI 1,701,963 monotherapy or combined with BI 3,011,441	Pan-KRAS	Solid tumors	NCT04835714	Phase 1	SOS1 inhibitor monotherapyor combined with MEK inhibitor	Boehringer Ingelheim	Terminated, no results posted	[93]
BI 1,701,963 monotherapy or combined with Trametinib	Pan-KRAS	Solid tumors	NCT04111458	Phase 1	SOS1 inhibitor monotherapy or combined with BRAF inhibitor	Boehringer Ingelheim	Active, not recruiting	[93]

* The reference is not the publication related to the specific clinical trials but describe the characteristics of the newly developed drugs. Some of the drugs do not have publications, whose features we get from the sponsors’ information releases

** QTX3034 was reported to have the ability to target the KRAS G12D and G12V (efficacy relatively lower), but in this clinical trial, it is only used for KRAS G12D targeting

*** Trials on monotherapy of FDA approved Sotorasib and Adagrasib for indications and usages exploration and MEK/RAF inhibitors are not included

****because there are well summarized reviews for newly development drugs, here just lists the drugs whose clinical trials were posted after 2023 to update the latest advances

**Table 2:** Clinical trials for combined therapy of Sotorasib and adagrasib

Therapy	Tumor type	NCT number	Phase	Mechanism	Sponsor	Status	Reference^*^
Sotorasib, Panitumumab and FOLFIRI	CRC	NCT06252649	Phase 3	KRAS^G12C^ inhibitor, PD-L1 monoclonal antibody in combination with chemotherapy	Amgen	Not yet recruiting	[94]
Combination of Sotorasib and Platinum	NSCLC	NCT05920356	Phase 3	KRAS^G12C^ inhibitor in combination with chemotherapy	Amgen	Recruiting	[95]
Combination of Sotorasib and Panitumumab	CRC	NCT05993455	Phase 2	KRAS^G12C^ inhibitor and EGFR inhibitor	Korea University Anam Hospital	Active, not recruiting	[96, 97]
Sotorasib plus Lenvatinib	NSCLC	NCT06068153	Phase 2	KRAS^G12C^ inhibitor and RTKs inhibitor	ETOP IBCSG Partners Foundation	Not yet recruiting	/
Ladarixin With Sotorasib	NSCLC	NCT05815173 (NCT05815186) ^**^	Phase 1/2^**^	KRAS^G12C^ inhibitor and CXCR1/2 inhibitor	NYU Langone Health	Recruiting^**^	/
Carfilzomib in combination with Sotorasib	NSCLC	NCT06249282	Phase 1	KRAS^G12C^ inhibitor and proteasome inhibitor	City of Hope Medical Center	Recruiting	[98]
Sotorasib followed by stereotactic radiation therapy	NSCLC	NCT06127940	Phase 1	KRAS^G12C^ inhibitor and radiation	Karolinska University Hospital	recruiting	/
Adagrasib combined with stereotactic radiosurgery	NSCLC brain metastases	NCT06248606	Phase 2	KRAS^G12C^ inhibitor and Radiosurgery	Hoosier Cancer Research Network	Not yet recruiting	[99]
Adagrasib and nab-Sirolimus	Solid tumors	NCT05840510	Phase 1/2	KRAS^G12C^ inhibitor and mTOR inhibitor	Mirati Therapeutics Inc.	Recruiting	[100, 101]
BMS-986,466 with Adagrasib with or without Cetuximab	Solid tumors	NCT06024174	Phase 1/2	KRAS^G12C^ inhibitor and SHP2 inhibitor with or without EGFR monoclonal antibody	Bristol-Myers Squibb	Active, not recruiting	/
Adagrasib in combination with Olaparib	Solid tumors	NCT06130254	Phase 1	KRAS^G12C^ inhibitor and PARP Inhibitor	M.D. Anderson Cancer Center	Recruiting	/
Adagrasib and Durvalumab	NSCLC and gastro-intestinal cancers	NCT05848843	Phase 1	KRAS^G12C^ inhibitor and PD-1 monoclonal antibody	M.D. Anderson Cancer Center	Withdrawn	/

* The references are not necessarily directly associated with the clinical trials we list here, but describe the outcomes of the earlier preclinical or clinical trials for the related therapeutic strategies

** The status of NCT05815186 is withdrawn because it is combined with NCT05815173

## Immune microenvironment characteristics and targeting strategies in KRAS mutant tumors

**Fig. 2:**
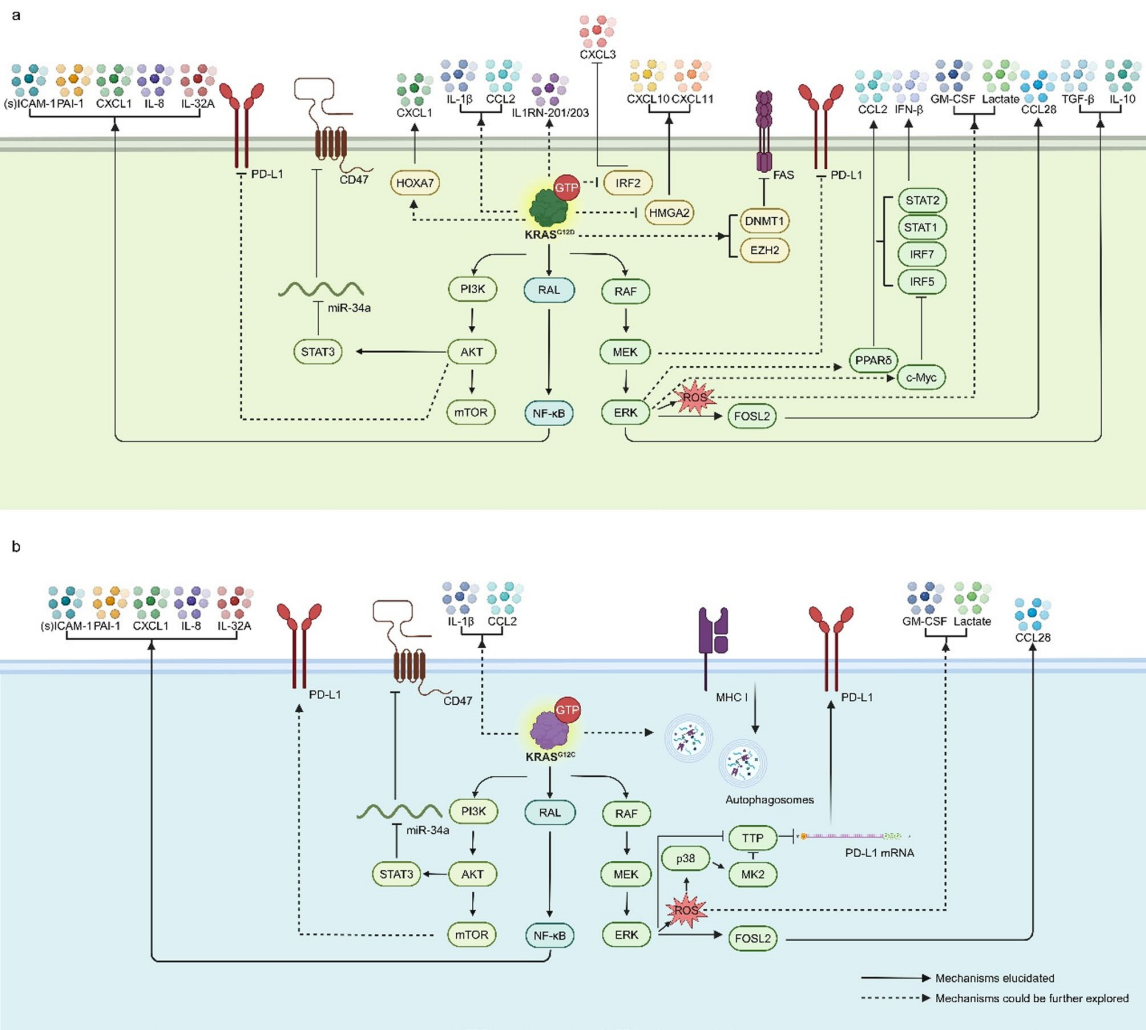
Comparison of KRAS^G12D^ and KRAS^G12C^ mediated immune-related signaling pathways. The mechanisms of regulating immune molecules by KRAS^G12D^ (**a**) and KRAS^G12C^ (**b**) mediated signaling transduction are depicted. Solid lines indicate established relationships, while dashed lines indicate mechanisms that warrant further exploration and detailed explanation

**Fig. 3:**
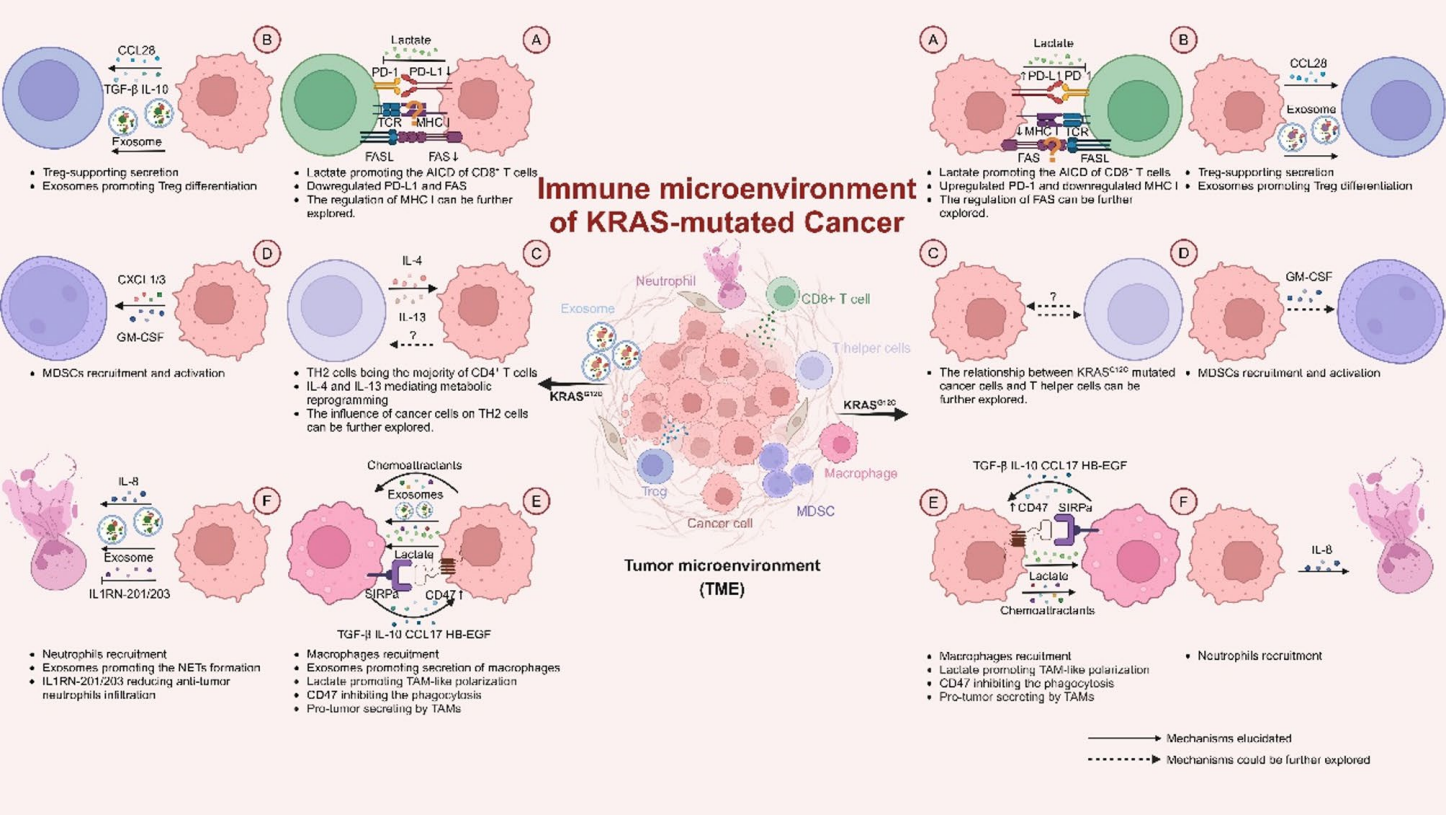
Comparison of KRAS^G12D^ and KRAS^G12C^ mediated immune microenvironment. The interactions between KRAS^G12D^-(left) and KRAS^G12C^-(right) mutated cancer cells with CD8^+^T cells(**A**), Tregs(**B**), T helper cells(**C**), MDSCs(**D**), macrophages(**E**), and neutrophils(**F**) are depicted. Solid lines indicate established relationships, while dashed lines indicate mechanisms that warrant further exploration and detailed explanation. The cell type is marked in the central sketch map

**Fig. 4:**
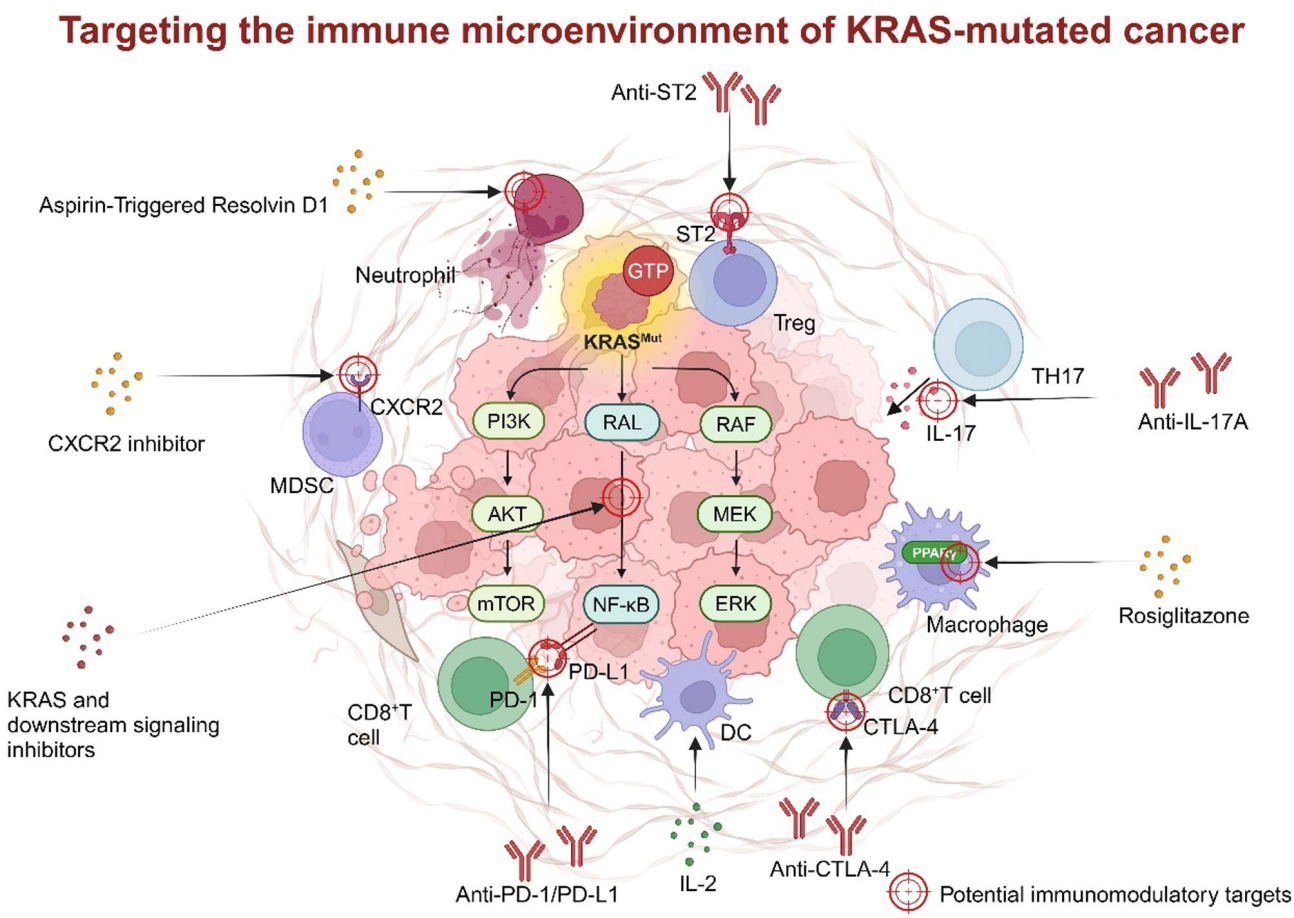
Targeting the immune microenvironment of KRAS-mutated cancer. The molecules on cells that could potentially or already be a target for immune microenvironment regulation therapy are highlighted, and corresponding drugs are linked to these targets by arrows

Evidence underscores the pivotal role of oncogenic KRAS in sculpting the immune microenvironment, with the majority of studies indicating that it predominantly fosters an immunosuppressive milieu [102, 103] and targeting KRAS shows the ability to remodel the TME [104] (Figs. 2 and 3). By elucidating the mechanisms KRAS modulates the microenvironment, we can generate novel insights that may inspire innovative therapies for KRAS-driven cancers (Fig. 4). We summarize recent immunotherapeutic clinical trials in Table 3 and for KRAS-mutated cancer. Here, we review the influence of oncogenic KRAS on various cell types in the tumor immune microenvironment and summarize the strategies to target the immune microenvironment of KRAS mutant cancer. It is important to note that while we have summarized the mechanistic role of KRAS mutations in regulating the immune microenvironment, clinical studies and some mechanistic investigations have only demonstrated an association between changes in the immune microenvironment and KRAS mutation status, without confirming a direct mechanistic link to KRAS. In these cases, KRAS mutations serve merely as a “biomarker” for such alterations, and their causal relationship still requires further research to be established.

### CD8^+^ T cells

#### CD8^+^ T cell dynamics in KRAS mutant tumors

As critical effectors of the adaptive immune response in the TME, the function of CD8^+^ T cells is significantly modulated by KRAS mutations. T cell infiltration is directly associated with KRAS mutations [105]. In NSCLC, the discoverers of AMG 510 demonstrated that the blockade of KRAS^G12C^ upregulated T cell infiltration, while MEK blockade did not produce this effect [56]. Conversely, KRAS^G12D^ reduced CXCL10/CXCL11 levels by downregulating HMGA2 levels, thereby reducing T cell infiltration [106]. Similarly, in PDAC, KRAS^G12D^ organoids mediate reduced T cell migration compared to KRAS^G12V^ organoids [107].

Alterations in the PD-1/PD-L1 axis impacted T cell immune responses and compromised the effectiveness of ICI therapies, subsequently influencing the prognosis and selection of immunotherapy in KRAS-mutated patients [108]. In lung adenocarcinoma (LUAD), KRAS mutation was reported to be associated with upregulation of PD-L1 [109], whose expression was highest in G12V, followed by G12C mutation [21]. However, in cases with the G12D mutation, PD-L1 expression was even lower than in patients with wild-type KRAS [21]. When expanding the scope to NSCLC, a study found that compared to G12A and G12C, there were more PD-L1-positive patients with G12D, G12V, and G13C mutations [110]. Cao et al. [111] reported that KRAS^G12D^ mutation was associated with positive but low PD-L1 expression, while other KRAS mutations were associated with high PD-L1 expression, in LUAD. More perplexing conclusions have been reached in colorectal cancer (CRC) regarding the relationship between KRAS mutations and PD-L1 expression, with findings spanning positive, negative, and unrelated statements [112–115]. Although high tumor mutational burden (TMB) in PDAC has been reported to be associated with favorable ICI efficacy and low KRAS mutation rates, the link was indirect [116]. The contradiction between some of the above conclusions indicated that the previous conclusion which stated that KRAS mutations lead to up-regulation of PD-L1 expression is oversimplified. It is worth noted that KRAS^G12D^ mutations have a distinctive role. Dividing patients into KRAS mutant and wild-type groups in the study obscures differences among different mutation types, which require attention in future clinical and basic research endeavors.

Studies have demonstrated that specific KRAS mutations modulate PD-L1 expression through distinct pathways. Specifically, the G12D and G12C mutations modulated PD-L1 expression via the MEK/ERK [117–119] and AKT/mTOR pathways [106, 120, 121]. The G12V and G12C mutations affected PD-L1 expression through the TTP downstream MEK pathway [122] while the G12V mutation could also modulate PD-L1 via the TGF-β and AKT pathways [123, 124]. The heterogeneity of KRAS mutational status limits the ability to draw definitive conclusions. Understanding more detailed mechanisms can help to have more and better choices in treatment. For example, in PDAC, the BET family member BRD4 is highly expressed. Inhibiting BET-mediated epigenetic regulation in mouse models also, in turn, affected KRAS signaling [125]. More elaborate subgroup and omics techniques analysis, rather than the idea of single-line regulation, were needed to reach a unified conclusion. To summarize with current knowledge, KRAS^G12C^ and KRAS^G12V^ may have the most positive relation with PD-L1 expression, and among so many kinds of mutations [126], KRAS^G12D^ has an outstanding possibility of being negatively associated with PD-L1 expression. However, that does not mean KRAS^G12D^ will not cause an immunosuppressive microenvironment, which could be induced by other pathways, and we will discuss it below.

In addition to PD-L1, another crucial factor influencing CD8^+^ T cell cytotoxicity is the Major Histocompatibility Complex (MHC) class I molecules. Downregulation of MHC I is acknowledged as one of the consequences of KRAS mutations [127]. Experiments conducted in a KRAS^G12C^ mouse model demonstrated increased MHC class I protein expression following treatment with MRTX849 [128]. Similar results were observed in the single-cell transcriptome profiling data of KRAS^G12D^ knockout pancreatic tumor xenografts [7]. At the protein level, autophagy was reported to be responsible for the reduction of MHC I in PDAC, and a similar phenomenon has been observed in many other KRAS mutant cancer cell lines, except for the CRC cell line [129]. Significantly, KRAS has been demonstrated to have the ability to regulate autophagy [130], with molecular mechanisms yet to be elucidated.

The FAS/FASL is another significant signaling pathway responsible for regulating the anti-tumor immune response by mediating apoptosis of CD8^+^ T cells. Mahadevan et al. [62, 103] conducted a series of studies uncovering that in KRAS^G12D^ pancreatic cancer mouse models. By genetic de-induction or drug inhibition of mutant KRAS, FAS expression was upregulated via DNA hypermethylation, promoting T cell infiltration. This finding was consistent with previous research in NSCLC [131], suggesting that in the case of KRAS^G12D^, downregulation of FAS was a key factor inhibiting the cytotoxic effect of T cells. KRAS mutation can assist cancer cells to counterattack T cells. In CRC, compared to wild-type KRAS, KRAS-mutated patients exhibited a relationship with abundance of CD8^+^ T cells and prognosis, and the CD8^+^ T cells showed high sensitivity of T cell activation-induced cell death (AICD) [132]. Lactate produced by KRAS-mutant tumor cells enters CD8^+^ T cells through MCT1 and regulated circATXN7 expression through histone lactylation. CircATXN7 inhibited NF-κB nuclear translocation, thus mediating this process [132, 133].

In conclusion, KRAS mutations significantly impact T cell infiltration, immune checkpoints, cytotoxicity, and cell death. This summary highlights the strong regulatory effect of KRAS mutations on T cells, the anti-tumor workhorses in the immune microenvironment. A noteworthy point is that KRAS mutations mediate immune suppression by targeting CD8^+^ effector T cells. However, while KRAS^G12C^ primarily exerts this effect through the upregulation of PD-L1 on tumor cells, the opposite result is observed in G12D tumors, which instead attenuate T cell cytotoxicity by downregulating FAS expression. This distinction significantly influences the selection of immunotherapies for patients with KRAS-mutant tumors. This highlights the importance of carefully considering grouping when conducting clinical and basic research to avoid masking these characteristics.

#### Strategies to target CD8^+^ T cells in KRAS mutant immune microenvironment

In discussions of targeting CD8^+^ T cells within the immune microenvironment, anti-PD-1/PD-L1 therapy is the primary focus. Liu et al. [105] reported enhanced efficacy of anti-PD-1/PD-L1 therapy in patients with KRAS mutations compared to those with wild-type KRAS, which aligned with findings from a large-scale meta-analysis [134]. It is noteworthy that the efficacy of anti-PD-1/PD-L1 therapy could vary among patients with different KRAS mutation subtypes, which will be discussed in detail in this section.

In KEYNOTE-042 and KEYNOTE-189 trials, patients with KRAS^G12C^ demonstrated better responses to immunotherapy than other KRAS subtypes. The KEYNOTE-042 trial evaluated immunotherapy versus chemotherapy for NSCLC [31]. Compared to those with KRAS wild-type (ORR: 29.1% vs. 21% for immunotherapy vs. chemotherapy), patients with KRAS mutations (ORR: 56.7% vs. 18.0% for immunotherapy vs. chemotherapy) demonstrated more significant ORR improvement from immunotherapy. Moreover, among patients with KRAS mutations, those with KRAS^G12C^ subtype exhibited superior immunotherapy outcomes (ORR: 66.7% vs. 23.5% for immunotherapy vs. chemotherapy) [31]. As for the KEYNOTE-189 trial [135] it evaluated chemotherapy combined with immunotherapy (immunochemotherapy) versus chemotherapy alone. It came out that KRAS^G12C^ subgroup had the highest ORR in immunochemotherapy (ORR: 50.0% vs. 18.2% for immunochemotherapy vs. chemotherapy), compared to those in the KRAS wild-type group (ORR: 47.6% vs. 10.9%) and other KRAS mutation group (ORR: 40.7% vs. 26.7%) [135]. In a retrospective study which included 273 NSCLC patients who received first-line systematic therapy [136], two combination therapies, chemotherapy combined with immunotherapy, and chemotherapy combined with anti-angiogenic therapy, were evaluated. KRAS-mutant NSCLC patients showed a significant benefit in efficacy when treated with first-line chemotherapy combined with immunotherapy, compared to first-line chemotherapy combined with anti-angiogenic therapy. Moreover, KRAS^G12C^ mutations, in comparison to other types of KRAS mutations, demonstrated a significant improvement in PFS [136]. The efficacy of anti-PD-1/PD-L1 therapy was diminished in patients with KRAS^G12D^ mutations [137] with a large-scale dataset confirming this finding [22]. Two retrospective studies have demonstrated that all types of KRAS mutations benefit from ICI therapy, with no difference in median progression-free survival (mPFS) between different KRAS mutation types [138, 139]. However, one of these studies found that patients with KRAS^G12C^ had better median overall survival (mOS) [139].

In summary, most studies have confirmed that among different KRAS mutation subtypes, KRAS^G12C^ demonstrates better efficacy with immunotherapy, while KRAS^G12D^ shows poorer response. A few studies report conflicting results, and there is a lack of data on the immune treatment efficacy for other KRAS mutation subtypes. Large-scale studies and meta-analyses are needed in the future to address these gaps. Future clinical research should place sufficient emphasis on reporting KRAS mutation subtype data. Subtyping is essential for conclusively determining the role of KRAS mutations in patient stratification. For example, KRAS^G12D^ is associated with primary resistance to anti-PD-1/PD-L1 therapy in NSCLC [106], highlighting the need for potential combination therapies or novel strategies.

The concept of relieving the inhibitory effect of KRAS mutations on T cells while simultaneously targeting immune checkpoints through combination therapy is highly appealing, considering the regulation mentioned above of T cells by KRAS. For instance, in the KRAS^G12C^ mouse model, the combination of anti-PD-1 and AMG 510 exhibited significantly improved effectiveness compared to monotherapy [56]. Combining the KRAS^G12D^ inhibitor MRTX1133 with anti-PD-L1 in pancreatic cancer produced comparable effects [62]. In a preclinical study on CRC, combining the KRAS^G12D^ inhibitor RMC-9805, RAS (ON) multi-selective inhibitor RMC-6236 and anti-PD-1 achieved 100% complete regressions, compared to 20% with anti-PD-1 alone and 60% with the combination of RMC-6236 with RMC-9805 [140]. Similarly, in PDAC, RAS(ON) multi-selective inhibitor RMC-7977 sensitized pancreatic tumors to immunotherapy, including CD40 agonist, anti-CTLA-4 and anti-PD-1 [141]. Autophagy inhibition also sensitized PDAC to dual ICB with anti-PD-1/CTLA4 antibodies [129]. Moreover, investigating the enhancement of therapeutic efficacy by combining ICI with conventional chemotherapeutic agents is highly worthwhile. For instance, in non-small cell lung cancer, combining anti-PD-L1 with docetaxel did not enhance efficacy [105], whereas combining anti-PD-L1 with paclitaxel resulted in increased efficacy [106].

**Table 3:** Clinical trials of immune-related therapy for KRAS mutant cancer

Therapy	Mutant type	Tumor type	NCT number	Phase	Mechanism	Sponsor	Status	Reference^*^
LY3537982 plus immunotherapy with or without chemotherapy	KRAS^G12C^	NSCLC	NCT06119581	Phase 3	KRAS^G12C^ inhibitor and PD-L1 monoclonal antibody with or without chemotherapy	Eli Lilly and Company	Recruiting	[84, 85]
Durvalumab plus Platinum-based chemotherapy in combination with Tremelimumab or not	STK11 or KEAP1 or KRAS mutations or co-mutations	NSCLC	NCT06008093	Phase 3	CTLA-4 monoclonal antibody and chemotherapy with or without PD-1 monoclonal antibody	AstraZeneca	Recruiting	[142]
Adebrelimab in combination with SHR-8068 and chemotherapy	STK11 or KEAP1 or KRAS mutations or co-mutations	NSCLC	NCT06335355	Phase 2/3	PD-1 and CTLA-4 monoclonal antibody	Shanghai Shengdi Pharmaceutical Co., Ltd	Not yet recruiting	/
Anti-CD38 with KRAS vaccine and Anti-PD-1	KARS^G12A/C/D/R/S/V or G13D^	PDAC and NSCLC	NCT06015724	Phase 2	As described	Georgetown University	Recruiting	/
Divarasib and Pembrolizumab with or without chemotherapy	KRAS^G12C^	NSCLC	NCT05789082	Phase 1/2	KRAS^G12C^ inhibitor and PD-1 monoclonal antibody with or without chemotherapy	Hoffmann-La Roche	Recruiting	[143]
TNG260 in combination with Pembrolizumab	Mutated STK11 with or without KRAS mutation	NSCLC	NCT05887492	Phase 1/2	HDAC1 inhibitor and PD-1 monoclonal antibody	Tango Therapeutics, Inc.	Recruiting	[144]
TG01/QS-21 vaccination	KRAS or NRAS mutation on codon 12/13	Multiple myeloma and smoldering multiple myeloma	NCT05841550	Phase 1/2	Cancer vaccine	Oslo University Hospital	Recruiting	[145]
ELI-002 7P	KRAS^G12D/R/V/A/C/S or G13D^ or NRAS^G12D/R/V/A/C/S or G13D^	PDAC and CRC	NCT05726864	Phase 1/2	Cancer vaccine	Elicio Therapeutics	Recruiting	[146]
AFNT-211	KRAS^G12V^	Solid tumors	NCT06105021	Phase 1/2	Engineered T cells targeting KRAS^G12V^	Affini-T Therapeutics, Inc.	Recruiting	[147, 148]
Adoptive T-cell therapy	KRAS^G12V^, with appropriate HLA class II match (DRB1*07:01).	Pancreatic cancer	NCT05389514	Expanded access	Engineered T cells targeting KRAS^G12V^	Providence Health & Services	Remaining open	/
NT-112	KRAS^G12D^	Solid tumors	NCT06218914	Phase 1	Engineered T cells targeting KRAS^G12D^	Neogene Therapeutics, Inc.	Recruiting	/
KRAS- EphA-2-CAR-DC in combination with ICIs	KRAS^G12C/D/V^	Solid tumors	NCT05631899	Phase 1	CAR-DC and ICIs	Chinese PLA General Hospital	Recruiting	/
ATP150/ATP152, VSV-GP154 and Ezabenlimab	KRAS^G12D/V^	PDAC	NCT05846516	Phase 1	Cancer vaccine and anti-PD-1 monoclonal antibody	Amal Therapeutics	Recruiting	/
Genetically engineered autologous T-cells in conjunction with vaccine	KRAS^G12D/V^	Solid tumors	NCT06253520	Phase 1	Engineered T cells targeting KRAS^G12D/V^ and cancer vaccine	National Cancer Institute (NCI)	Recruiting	[149]
FH-A11KRASG12V-TCR	KRAS^G12V^	Solid tumors	NCT06043713	Phase 1	Engineered T cells targeting KRAS^G12V^	Fred Hutchinson Cancer Center	Recruiting	[150]
YK0901 cells	KRAS^G12V^	Solid tumors	NCT05933668	Phase 1	Engineered T cells targeting KRAS^G12V^	Peking University	Not yet recruiting	/
Botensilimab plus Balstilimab and fasting mimicking diet plus vitamin C	KRAS mutations	Colorectum adenocarcinoma	NCT06336902	Phase 1	CTLA-4 and PD-1 monoclonal antibody	University of Southern California	Not yet recruiting	[151]

* The references are not necessarily directly associated with the clinical trials we list here, but describe the outcomes of the earlier preclinical or clinical trials for the related therapeutic strategies

### CD4^+^ T cells

#### CD4^+^ T cell dynamics in KRAS mutant tumors

CD4^+^ T cells represent another crucial component of anti-tumor immunity due to their ability to regulate immune responses, warranting significant attention [152]. FACS and mIHC analysis of KRAS^G12D^ mutant PDAC tumors revealed that approximately 50% of the TME consisted of CD45^+^ cells, with 20% being CD3^+^ cells, predominantly of the CD4^+^ subtype [153]. Upon recognition of antigen-presenting cells, CD4^+^ T cells polarizing into distinct helper T cell subsets, including TH1, TH2, TH17, Treg, etc [152]. The effectiveness of the anti-tumor immune response depends on maintaining a balance between anti-tumor immune T cells (TH1 and CD8^+^) and tumor-promoting T cells (Treg, TH2, TH17) [154]. In the KRAS-mutated PDAC mouse model, ablation of CD4^+^ T cells reversed the formation of a suppressive immune microenvironment, including the following suppressive cytokines and MDSCs recruitment [155]. This finding suggests that the role of CD4^+^ T cells in remodeling the immune microenvironment extends beyond adaptive immunity, and the ratio of CD4^+^ T cell subgroups in KRAS-mutated cancer is dysregulated.

Tregs had a crucial role in the formation of tumor immunosuppressive microenvironment. In CRC, clinical samples containing at least one type of KRAS mutation showed increased infiltration of CD4^+^Foxp3^+^Tregs [156]. In NSCLC, tumor-derived exosomes from patients with any KRAS mutation type induced peripheral blood CD4^+^ T cell differentiation into Tregs [157]. The evidence confirmed the robust and direct regulation of Treg recruitment and differentiation by mutant KRAS. Kim et al. [158] identified ST2 as a unique molecule expressed by Treg in KRAS^G12D^ mutant lung cancer. Targeting ST2 resulted in not only the depletion of Tregs but also the following of M2 cells [158]. Furthermore, in the case of KRAS^G12D^ in CRC [159] or KRAS^G12V^ in PDAC [160], the upregulation of MEK/ERK/IL-10 and MEK/ERK/TGF-β promoted the Tregs differentiation and inhibited T cell activation. In PDAC with G12C or G12D mutations, FOSL2, another downstream target of MEK/ERK, was found to recruit T cells via increased secretion of CCL28 [161]. Those conclusions further confirm that the oncogenic activation of the MAPK pathway directly promoted Tregs infiltration.

In KRAS^G12D^ mutant PDAC, another study demonstrated that KRAS^G12D^ elimination increased the infiltration of CD4^+^ and CD8^+^ T cells while decreasing the proportion of PD-L1-positive myeloid cells [103]. While the increased CD4^+^ T cells were predominantly suppressive, they eventually exhibited an overall positive tumor suppressive effect. The researchers interpreted the increased presence of suppressive CD4^+^ T cells after KRAS^G12D^ elimination as a natural constraint on the cytocidal effect of CD8^+^ T cells. It is important to note whether this effect occurs in the context of other KRAS mutations, as it may partially contribute to KRAS inhibitor resistance mechanisms.

There has been limited focus on TH1 cells, which are known as tumor suppressors within the CD4^+^ T cell population. Analysis of public data suggested that KRAS mutant CRC exhibits downregulated TH1 cells and IFN-γ pathways, and the extent of the effect was influenced by CMS typing [162]. However, detailed KRAS mutation typing is lacking, making it uncertain whether specific KRAS mutation types act as confounders. Understanding the frequency and characteristics of TH1 cells in the context of different KRAS mutations, as well as the mechanisms driving their recruitment, differentiation, and function within tumors, is of significant importance. This area warrants further investigation.

The secretion of cytokines achieves the primary function of various helper T cells. During the PanIN stage of the KRAS^G12D^ PDAC mouse model, TH2 cells occupied the highest ratio of CD4^+^ T cells [153]. The secreted IL-4 and IL-13 from TH2 cells upregulated c-Myc and promote metabolic reprogramming, thereby promoting tumorigenesis [153]. Interestingly, IL-22 was closely related to the c-Myc pathway in CRC cell lines with KRAS^G13D^ mutation, where it cooperated with KRAS mutations to promote tumor cell proliferation [163]. This cytokine is primarily secreted by TH17 cells and γδ T cells, implicated in cancer promotion.

Moreover, IL-22 was found to upregulate an immunosuppressive inflammatory microenvironment and induce stemness in KRAS^G12D^ mutant lung cancer mice, possibly by mediating the activation of STAT3 and ERK pathway [164]. Another major cytokine secreted by TH17 cells is IL-17, known for its pro-inflammatory properties. IL-17 has been implicated in tumorigenesis in both KRAS^G12D^ NSCLC [165] and PDAC [166] primarily due to its pro-inflammatory properties, including recruitment to Gr-1^+^ CD11b^+^ MDSCs [167]. Moreover, IL-17 and IL-22 have been implicated in mediating resistance against MEK inhibitors [119]. However, the results of a clinical trial of the MEK inhibitor selumetinib in KRAS-mutated advanced NSCLC patients were perplexing, as high TH17 levels at baseline and increased TH17 levels after treatment were associated with response and better PFS, respectively [168]. This may be related to the heterogeneity of the immune response observed across different types of KRAS mutations, warranting further investigation. While the mechanism of action of these cytokines has been elucidated, the recruitment mechanism of their secretory cells remains to be explored, which is crucial for targeted therapy.

#### Strategies to target CD4^+^ T cells in KRAS mutant immune microenvironment

In the face of dysregulated cell proportions, down-regulating Tregs is the primary strategy for targeting CD4^+^ cells. Research has demonstrated that both KRAS inhibition [160] and MEK inhibition [159, 161] effectively suppress Treg recruitment. ST2 monoclonal antibody has also shown efficacy in non-small cell lung cancer with KRAS^G12D^ mutations [158]. Exploring the use of ICI drugs following Treg down-regulation to enhance T cell functionality within the immune microenvironment is worthwhile.

Current cytokine therapies primarily involve antibody-based approaches [169]. Therapeutic interventions targeting cytokines in KRAS mutant tumors are uncommon, although studies have demonstrated the potential of IL-17 A monoclonal antibodies in combination with MEK inhibitors and anti-PD-L1 therapy [119]. Given their role in modulating the dysregulated immune microenvironment, cytokine-targeted therapies warrant further investigation.

### Innate immunity cells

#### Neutrophil dynamics in KRAS mutant tumors

Tumor-associated neutrophils exhibit heterogeneity, generally manifesting pro- or anti-tumor phenotypes [170]. Usually, KRAS mutations promoted neutrophil infiltration, typically associated with a pro-tumor phenotype [171–173]. IL-8, a critical chemokine recruiting neutrophils and a clearly defined RAS target [174, 175], was upregulated in various KRAS mutant tumors [176–178] primarily through the NF-κB pathway [179–181], which is highly likely to be the primary driver of neutrophilic infiltration. CRC exhibited a unique profile among KRAS mutant tumors, with KRAS mutations negatively associated with neutrophil infiltration [162]. However, CRC cancer cells with KRAS^G12D^ mutations transferred mutated KRAS protein to neutrophils via exosomes, thereby promoting the pro-tumor factor neutrophil extracellular trap (NET) by upregulating IL-8 expression [182].

The upregulation of neutrophils played a multifaceted role by interacting with other cells in the microenvironment. In KRAS^G12V^ liver cancer, neutrophils activated HSCs to played pro-tumor role [183]. However, in KRAS^G12V^ ovarian cancer, neutrophils supported the anti-tumor immunity of T cells [184], possibly through GM-CSF upregulation [185]. In KRAS^G12D^ intrahepatic cholangiocarcinoma, IL1RN-201/203 upregulation reduced neutrophil infiltration and the proportion of pro-tumor neutrophils [173]. Proper manipulation of this equilibrium could facilitate therapeutic goals by reprogramming the microenvironment.

The relationship between various KRAS mutation sites and neutrophil pro- or anti-tumor phenotypes in different tumor types is somewhat understood. Yet, the underlying reasons for these different phenotypes remain unclear. Identifying the origin of these differences is crucial for understanding the relationship between KRAS and neutrophils, potentially unlocking targeted intervention strategies.

#### Macrophage dynamics in KRAS mutant tumors

Macrophages are well-studied subjects in innate tumor immunity and play a cancer-promoting role in the microenvironment of KRAS mutant tumors. The infiltration of macrophages was downregulated by KRAS^G12C^ inhibitor MRTX849 in KRAS^G12C^ NSCLC [128]. GM-CSF physiologically promotes macrophage production and is potentially a critical actor for the abnormal increase in macrophages [186]. In CRC, KRAS^G12C/D/V^ mutations resulted in ROS accumulation, leading to the upregulation of lactate and GM-CSF, thus recruiting macrophages and transforming them into more tumor-supportive tumor-associated macrophages (TAM)-like cells that secreted higher levels of IL-10, CCL17, and TGF-β1 [187]. A similar conclusion has been demonstrated in KRAS mutant PDAC [188]. Additionally, Miller et al. [189] highlighted the critical role of the deficiency of tumor suppressor DAB2IP in KRAS mutant colon cancer in causing the release of inflammatory mediators via NF-κB, suggesting it as a fundamental reason for macrophage recruitment. In KRAS^G12D^ mutant lung cancer, nitric oxide (NO•) was also involved in the recruitment and regulation of macrophages through the secretion of inflammatory mediators [190]. In pancreatic acinar cells, KRAS^G12D^ was responsible for upregulating soluble ICAM-1, a macrophage chemoattractant [191, 192]. Besides the cytokines mentioned above, the PPARδ-CCL2/CCR2 axis has also been upregulated in KRAS^G12D^ PanIN lesions, contributing to the recruitment of macrophages and MDSCs [193]. Similarly, various KRAS mutation cell lines exhibited significantly higher expression of CCL2 compared to both wild-type cancerous and healthy cell lines [194].

The recruited macrophages were found to support the growth of KRAS^G12D^ lung cancer cells in a mTOR-dependent way [195]. Additionally, the senescent-associated secretory phenotype contributed to TAMs promoting KRAS-mutated lung cancer [196]. Germline knockout of the traditional tumor suppressors p16 or p21, which were markers and effectors of senescence, inhibited tumor progression in the KRAS^G12D^-mutated lung cancer mouse model [197].

Macrophages exhibit M1 and M2 polarization states, these two states have opposite functions. During the initiation of KRAS mutant PDAC, M1-type macrophages accelerated tumorigenesis by forming an inflammatory microenvironment [191, 192]. TAMs usually assume an M2-like phenotype to shape a microenvironment conducive to tumor progression, with immunosuppressive and multiple cancer-promoting characters [198, 199]. Additionally, M1-type macrophages in early lesions of intestinal serrated adenomas with KRAS^G12D^ were considered a protective factor, similarly in KRAS mutant CRC [156, 200]. In KRAS^G12D^ mutant pancreatic epithelium organoids, macrophages shifted towards M2, promoting cancerous changes [201]. Similarly to neutrophil activation, KRAS^G12D^ PDAC cells could transfer mutant KRAS protein to macrophages via exosomes, thereby promoting their M2-like phenotype through a STAT3-dependent pathway [202]. M2-like macrophages secreted heparin-binding epidermal growth factor-like growth factor (HB-EGF), activating EGFR and promoting KRAS oncogenic signaling in KRAS^G12D^-mutated PDAC [203]. The ongoing debate regarding the effects of M1-type macrophages on cancer cells indicates that more researches are needed to clarify the precise role of macrophages or inflammation in different cancer initiation. Hu et al. [204] identified CD47 as a major effector of KRAS-mediated innated immune evasion. The upregulation of CD47 in cancer cells contributed to decreased phagocytosis by macrophages via activation of the suppressive receptor SIRPa. This downregulation of function could be understood as a form of macrophage polarization mediated by KRAS mutation.

#### Dendritic cell (DC) dynamics in KRAS mutant tumors

DCs primarily function as anti-tumor immune cells by presenting antigens [205]. KRAS mutant PDAC [206] and LUAD [172] exhibited reduced DC infiltration, while MRTX849 [128] or analogous drug MRTX1257 [207] treatments could reverse that effect, indicating a negative correlation between KRAS mutations and DC presence. Additionally, biopsies from the KRAS mutant CRC cohort displayed a higher CD1a^+^DC-LAMP^+^ DC ratio, which indicated more infiltration of immature DCs [208]. However, in PDAC, KRAS inactivation showed minimal impact on DCs [209] suggesting a weaker link between KRAS mutations and DC alterations in this context.

Dendritic cells play a crucial role in adoptive cell therapy, and clinical studies are currently investigating their performance in treating KRAS-mutant tumors (NCT05631899). IL-2 expression is associated with OS of PDAC patients and positively correlated with CD8^+^ T cells, activated NK cells, and DCs, while negatively correlated with M0 macrophages. In adoptive cell therapy, using DCs treated with IL-2, PBMCs, KRAS^G12D^_1−23_ peptide, or tumor lysates resulted in the increase of CD8^+^ T cells and decrease of TH1 cells, TGF-β and IL-10. Additionally, it upregulated PD-1 expression, suggesting potential therapeutic value in combination with anti-PD-1 therapy [210].

Although research on DCs in the context of KRAS mutations is limited, and their recruitment and effector mechanisms remain undefined, existing studies consistently indicate a correlation with KRAS mutations. Given DCs’ indispensable role in initiating and regulating innate and adaptive immunity, they warrant further attention [205].

#### Myeloid-derived suppressor cell (MDSC) dynamics in KRAS mutant tumors

MDSCs constitute major innate immune-suppressive cells within the immune microenvironment. They directly inhibited T cells and engaged in various pro-cancer activities [211]. In CRC, KRAS^G12D^ downregulated the expression of IRF2, leading to the upregulation of CXCL3, which bound to CXCR2 on the surface of MDSCs, facilitating their recruitment and activation. The recruited MDSCs directly inhibited the immune response of CD8^+^ T cells and reduced the sensitivity to anti-PD-1 therapy [212]. The CXCL1 and GM-CSF, upregulated by KRAS^G12D^ as mentioned above in macrophage recruitment, also contributed to the recruitment of MDSCs in PDAC [213, 214], concurrently inhibiting the infiltration of CD8^+^ T cells [215]. Inhibiting KRAS [128] MEK [216] and ERK [217] has been shown to effectively decrease the infiltration of MDSCs, indicating that MDSC recruitment benefits from the canonical MAPK signaling pathway. Although the current research evidence is not rich, it underscores the critical role of MDSCs in modulating immune responses within the immune microenvironment mediated by KRAS mutations. Further investigation into the recruitment and activation mechanisms of MDSCs is likely to elucidate the complex interplay between cancer genetics and immune evasion, potentially paving the way for breakthroughs in treatment strategies.

#### NK cell dynamics in KRAS mutant tumors

NK cells function as tumor killers, possessing the ability to target and eliminate cancer cells within the immune microenvironment [218]. PanINs demonstrated significant resistance to NK cell-mediated cytotoxicity during tumorigenesis by secreting higher levels of IL-6 and exhibiting reduced CD44 expression. IL-6 was essential for MAPK activation; even with oncogenic KRAS mutations, the absence of IL-6 halted cancer development in PDAC mouse models [219] and was shown to prevent initiation while enhancing the progression of lung cancer driven by KRAS mutations [220]. The proliferative activity, cell-killing capacity, and IFN-γ secretion capability of NK cells were significantly reduced in pancreatic KRAS^G12D^ mice compared to wild-type mice [221]. Evidence suggested that KRAS mutations could inhibit NK cell infiltration [104, 222, 223]. For instance, KRAS^G12D^ suppressed NK cell infiltration by upregulating Myc and inhibiting the downstream I-type IFN pathway in PDAC [223]. And there was similar mechanism in KRAS^G12C^ mutant lung cancer [19]. Additionally, in CRC, KRAS mutations was related to high proportion of CD56_bright_ NK cells infiltration, which might drive a poor response to ICIs, even in the CD8^+^ T cell-high TME [224].

The success of CAR-T therapy has redirected attention towards NK cells, with the recognition that they can be harnessed for similar therapeutic objectives [218]. It emphasizes the critical need to comprehend NK cell mechanisms within tumors, especially the nuances across varying contexts, such as against the backdrop of KRAS mutations.

#### Summary of innate immune microenvironment of KRAS mutant cancer

Research on innate immune cells, unlike that on T cells, remains less extensive, a situation that applies not only to KRAS-mutated tumors, with even fewer studies focusing on subtype-specific differences. As research deepens and technology advances, developing new methods to target innate immune cells is inevitable. Basic research has revealed that each component of the immune microenvironment plays a regulatory role in tumor development, suggesting that optimal targeting of the immune microenvironment should be multifaceted and balanced. As part of precision immunomodulation, deciphering the innate immune microenvironment across different subtypes may offer insights into the immune therapy discrepancies observed among KRAS-mutant subtypes. This approach holds promise for identifying potential targets for precise immune microenvironment regulation.

#### Potential and challenges of immune modulators beyond immune checkpoint inhibitors

By reviewing the immune microenvironment characteristics of KRAS-mutant tumors comprehensively, it is clear that, in addition to the existing ICIs, there are many promising non-checkpoint inhibitor approaches for precision immune microenvironment modulation. Targeting innate immune cells presents a viable strategy, but it potential toxicity significantly influences its development and application. In a KRAS^G12D^-mutated lung adenocarcinoma mouse model, Aspirin-Triggered Resolvin D1 demonstrated efficacy by reducing the ratio of neutrophils to lymphocytes and inhibiting tumor growth [225]. The PPARγ agonist rosiglitazone promoted M1 polarization of macrophages and reduced IL4 expression, thus inhibiting the formation of intestinal serrated adenomas in KRAS^G12V^ mice [200]. A CXCR2 inhibitor targeting MDSCs, combined with PD-L1 inhibition, has been shown to significantly inhibit the metastasis of KRAS-mutated CRC cells in mouse models [213]. And an interesting study reported that the use of KRAS pathway inhibitors, such as KRAS^G12C^ inhibitors or SHP2 inhibitors, led to an upregulation of MDSCs infiltration, thereby suppressing anti-tumor T cell immunity [226]. This highlights the possible impact of resistance mechanisms to KRAS inhibitors on ICI therapy, suggesting that KRAS inhibition could potentially diminish the efficacy of immunotherapy. In this study, the combination of KRAS pathway inhibitors and CXCR2 inhibitors demonstrated therapeutic efficacy, suggesting that future strategies could incorporate CXCR2 inhibitors into KRAS inhibitor and immune therapy combinations to enhance therapeutic outcomes. Assessing the specificity of the therapeutic effect remains a paramount concern before clinical trial initiation.

Alternatively, targeting immune modulating pathways in cancer cells presents another strategy. Hammoudeh et al. [180] employed the Snail-p53 binding inhibitor GN25 to treat A549 cells, observing the activation of immune response pathways related to neutrophils and T-cells in the transcriptome. The binding of Snail to p53 occurs downstream of KRAS oncogenic activation [227] with both factors associated with immune modulation [180]. Inhibition of cytokine secretion, such as IL-6 and CCL5, using JAK/TBK1/IKKε inhibitors has also been demonstrated as a practical approach [228]. Although lacking validation regarding the inclusion of immune cells, they offer valuable insights for identifying potential targets. Unlike targeting T cell immunity, this type of research is still at a relatively early stage, and in addition, valuable discoveries may play an essential role in improving therapeutic efficacy with combinations of drugs.

Furthermore, several potential therapeutic strategies have yet to be validated in the context of KRAS mutations. However, based on existing preclinical evidence, these strategies show promise in modulating the immune microenvironment in KRAS-mutant tumors. For instance, as previously mentioned, TAMs have been shown to regulate the proliferation of KRAS^G12D^ mutant lung cancer through mTOR signaling, its reprogramming holds potential for enhancing the therapeutic efficacy in this type of tumor [146]. Recent study has confirmed that in PDAC, the combination of gemcitabine, PD-L1 monoclonal antibodies, and mTOR inhibitors outperforms any of the single agents or dual-agent combinations above in terms of therapeutic efficacy [229]. The role of mTOR inhibitors worked through the promotion of glycolysis in M2-type TAMs, thereby suppressing their tumor-promoting effects. This shift in metabolic regulation led to inhibition of tumor cell proliferation and a reduction in tumor cell glycolysis [229]. Similarly, neutrophil reprogramming is a promising therapeutic approach [230]. Recent reports have suggested that iron regulation can inhibit the formation of NETs, thereby enhancing the efficacy of cancer immunotherapy [230]. Given the upregulation of NETs in KRAS^G12D^ CRC [182] this combined treatment strategy warrants investigation in such tumors. Cytokine inhibitor-based therapies targeting immune microenvironment modulation are also under exploration [231]. For example, a study has shown that IL-1β blockade in KRAS^G12D^ mutant LUAD mouse models can reduce neutrophil and MDSCs infiltration while increasing T-cell infiltration levels [232].

## Specific immune microenvironment in the case of Co-mutations and relative therapeutic challenges

**Fig. 5:**
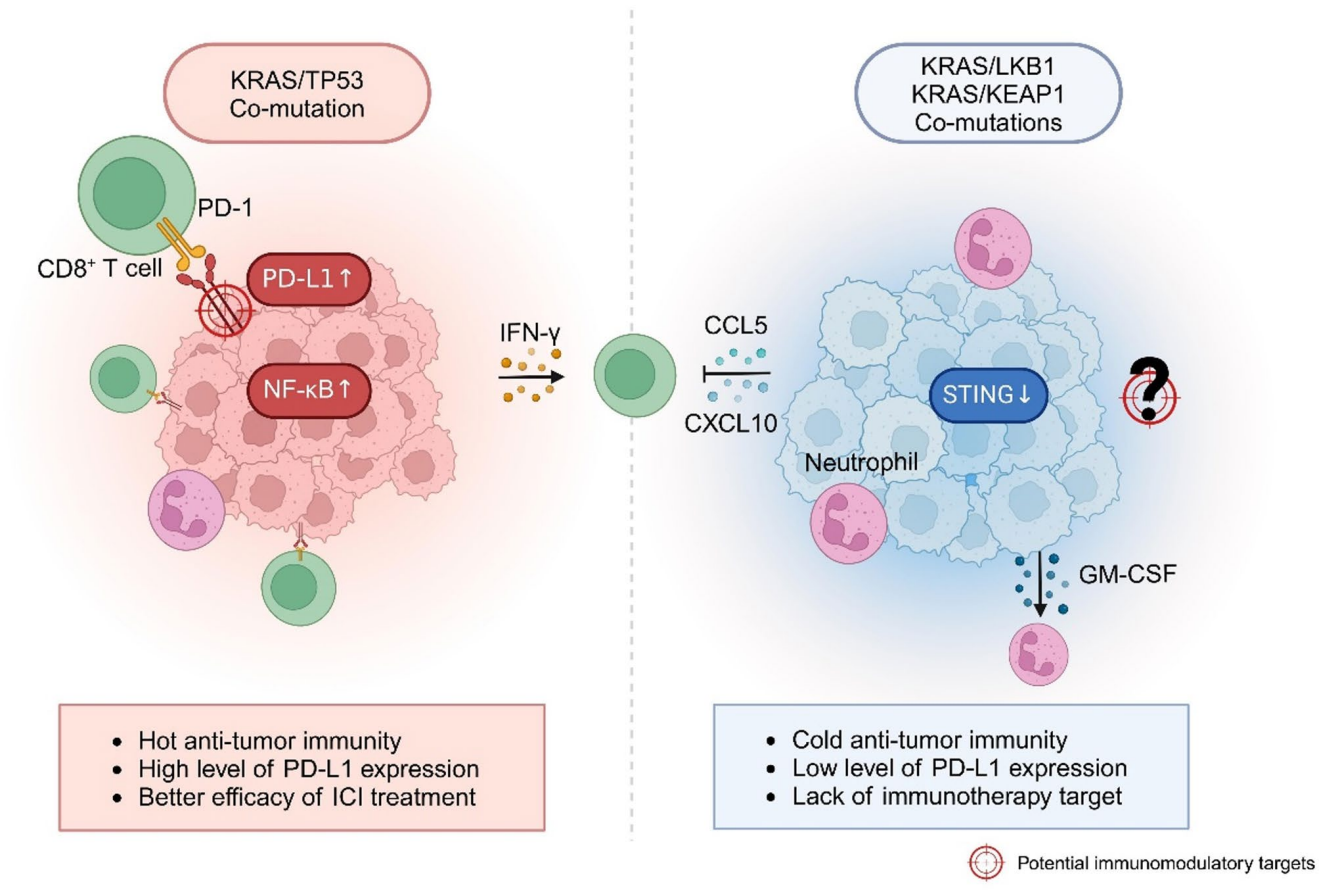
Comparison of the immune microenvironment across different co-mutations. Differences in immune cell infiltration and immune-related pathway statuses between KRAS/TP53 co-mutation (left) and KRAS/LKB1 or KRAS/KEAP1 co-mutations (right) are depicted. Arrows mark the changes in immune-related signaling pathways, and the available immunotherapy target is highlighted

After summarization of the immune microenvironment of KRAS mutant cancer, we highlighted the heterogeneity between different types of KRAS mutation. In an era where precision medicine is increasingly pivotal, we underscore the significance of precise subtyping in diagnosis and treatment, particularly when confronting tumors characterized by high heterogeneity. In addition, recent studies have noticed that co-mutations with KRAS also play a pivotal role in the oncogenesis and immune landscape of tumors, making them a significant focus for cancer research and therapy. The presence of co-mutations and research into their therapeutic strategies, to some extent, compensates for the limitations of classification based solely on KRAS mutation types (Fig. 5). Co-mutations are associated with prognosis and drug sensitivity [233, 234], especially the response to checkpoint inhibition [235]; a study involving 330 advanced KRAS mutant lung cancer patients showed that patients with KRAS/KEAP1/NFE2L2 co-mutation had shorter OS and worse response to both platinum-based chemotherapy and immune therapy [236]. The mechanisms involved are still unclear, but analysis of extensive clinical data suggested that alterations in the immune microenvironment played a significant role. Certain co-mutations, like TP53, lead to a “hot” immune microenvironment and are more responsive to immunotherapy. Others, like LKB1 and KEAP1, are associated with a “colder” microenvironment and poorer outcomes [237, 238]. Skoulidis et al. [239] identified three clusters within LUAD RNA-seq data characterized by TP53, LKB1, and CDKN2A co-mutations rather than different KRAS mutations, while Liu et al. [240] concluded two clusters of KRAS mutant LUAD with multi-omics analysis, one characterized by LKB1/KEAP1 co-mutation. Both studies highlighted the predominance of co-occurring genetic events in the biological diversity of KRAS mutant LUAD.

Scheffler et al. [241] suggested that there was a partial association between co-mutated genes and distinct mutant types of KRAS after researching several co-mutations in KRAS mutant NSCLC. Still, only a few of the co-mutations showed significant differences. In contrast, another research showed that in multiple kinds of cancer, LKB1 or KEAP1 co-mutation tended to be accompanied by KRAS^G12C^ rather than other types of KRAS mutations [242]. To surmise with current findings, there may be subtle gaps in downstream pathway activation for different kinds of KRAS mutations, but only co-mutations trigger gaps at the transcriptome level. Despite the genes with top mutant rates, including TP53, LKB1, and KEAP1 ^241^, an increased number of mutations in the above genes often means a worse prognosis [234]. Therefore, the importance of co-mutations in prognosis and treatment selection should be realized.

### Immunotherapy responses in different co-mutation backgrounds

STK11 and KEAP1 co-mutations with KRAS are primarily observed in lung cancer, as these two genes are rarely mutated in other cancers with high KRAS mutation rates [243, 244]. TP53 co-mutation with KRAS is more common, as TP53 is the most frequently mutated gene across all cancers [245]. In NSCLC, patients with TP53 and KRAS co-mutations showed significantly improved response rates and median survival compared to those with KRAS mutations alone [246]. However, in LUAD, patients with co-mutations of LKB1 or KEAP1 and KRAS had a poor prognosis with ICI monotherapy, a phenomenon not observed in KRAS wild-type patients [247]. In PDAC, patients with KRAS and TP53 co-mutations showed improved OS when treated with chemotherapy combined with anti-PD-1 therapy compared to chemotherapy alone d [248]. In contrast, in metastatic colorectal cancer, co-mutations of RAS/BRAF and TP53 were associated with poor prognosis [249]; but no studies have yet assessed whether patients with these co-mutations could benefit from immunotherapy. More multicenter, large-scale studies and future meta-analyses are needed to systematically compare and summarize these findings.

### KRAS/TP53 co-mutation

TP53 mutation itself can drive carcinogenesis in multiple tumor cells as one of the most classical tumor suppressors [245]. It shows the highest co-mutant rate in KRAS-mutated NSCLC [241]. When treated with ICIs, patients with KRAS/TP53 co-mutation showed better prognosis than those with only KRAS mutation, associated with the significantly highly expressed PD-L1 [238, 250, 251]. Simultaneously, the highest proportion of CD8^+^ T cells in KRAS/TP53 co-mutated LUAD was higher than those with only KARS- or TP53-mutation [18]. Interestingly, even ICI treatment in TP53-mutated patients with low PD-L1 expression remained better performance [238], suggesting additional mechanisms behind the immune regulating driven by the co-mutation and making KRAS/TP53 co-mutation a predictive role beyond PD-L1 expression [18]. Pathway enrichment analysis revealed several pathways associated with activating anti-tumor immunity and immune tolerance/escape in KRAS/TP53 co-mutated LUADs [239] such as IFN-γ signaling [250]. PD-1 was also downregulated in CD8^+^ T cells in CRC when there was KRAS/TP53 co-mutation [252]. Whether this is the specific characteristic of CRC or a kind of negative feedback remains unclear, yet it is noteworthy for selecting PD-1 or PD-L1 inhibitors. “Hot” immune microenvironment formation could be explained by the combination of activation of multiple pro-inflammatory pathways, especially NF-κB in tumor cells, and the increasing number of neoantigens caused by higher mutational burden, which was the special combination under the background of KRAS/TP53 co-mutation [23, 253, 254]. It is important to note that the KRAS^G12D^/TP53 co-mutation may be a particular case, as studies have identified a lower level of PD-L1 expression and a lower degree of immune infiltration [222]. In PDAC, the transcriptomic changes orchestrated an inflammatory immune microenvironment with the activation of several innate immunity-related pathways [255], possibly due to the high rate of G12D mutation. Further research, especially meta-analysis, is needed to reconcile varying findings. To summarize, the KRAS/TP53 co-mutation drives the immune microenvironment “hot”, characterized by activating multiple pro-inflammatory and immune escape pathways.

KRAS/TP53 co-mutation is special because it made immunotherapy more effective (as assessed by PFS) than either mutation or both wild-type [256]. From a TP53 perspective, it was one of the most effective immunotherapies when co-mutated with KRAS compared to other co-mutations [256]. Notably, the responsibility to ICIs by KRAS/TP53 co-mutated cancer is not absolute. There were still patients with the KRAS/TP53 co-mutation who cannot benefit from ICI treatment [238]. What is more, in samples without KRAS co-mutation, the level of PD-L1 expression and IFN-γ activation varied between populations with TP53-missense-mutation and those with TP53-nonsense-mutation [257]. Similarly, Pan et al. [258] found if the TP53 mutation states alone was influenced the prognosis in PDAC.

Current therapeutic studies of ICIs targeting KRAS/TP53 co-mutations are focused on lung cancer. Other tumors are waiting assessment, including colorectal cancer [259], intrahepatic cholangiocarcinoma [260], biliary tract cancer [261], high-grade appendiceal mucinous neoplasm [262], and gastric cancer [263]. Although KRAS/TP53 co-mutations have demonstrated an excellent response to ICIs compared to other co-mutations, we are still looking forward to better therapeutic effects and treatment options. Perhaps the combination of targeted agents and immunotherapy could work wonders in this type of tumor. For instance, in the KRAS/TP53 co-mutation-driven lung cancer mice model, the combination of MEK inhibitor and anti-PD-(L)1 promoted the immune response better than a single agent [264].

It’s vital to note that some of the genetically engineered mice models for PDAC tumors carry co-mutations in KRAS and TP53 [265]. Despite the high mutation rates of KRAS and TP53 in PDAC within the real-world patient population, some patients do not exhibit these co-mutations. Therefore, researchers should exercise caution in conducting and reporting their studies to ensure the research represents a broader patient population.

### KRAS/LKB1 co-mutation

LKB1, or STK11, works upstream of AMPK. AMPK is a metabolic switch regulating intracellular glucose and lipid metabolism, and links serine biosynthesis to tumorigenesis [266]. It is among the most common mutant tumor suppressors in lung cancer, especially in NSCLC [267, 268]. The single-gene deficiency of LKB1 alone could not initiate tumorigenesis; however, LKB1-deficient KRAS mutant LUAD cases exhibited more rapid development, more frequent metastasis than TP53- or CDKN2A-deficient ones [269]. And contrary to KRAS/LKB1 co-mutation, LKB1 co-mutation showed diminished efficacy of PD-1/PD-L1 Inhibition [247, 270]. Numerous mechanisms underlied this, including effects on invasion and metastasis promoting [271] metabolic rewiring [272], extracellular matrix remodeling [273], and angiogenesis [274]. Beyond those, immune reprogramming could be important because of its well-studied mechanisms and promising problem-solving ability. KRAS/LKB1 co-mutated LUAD patients exhibited resistance to ICIs due to low immune engagement, characterized by low infiltration of CD3^+^, CD8^+^, CD45RO^+^ T cells, CD68^+^ macrophages and mature DCs, high infiltration of neutrophils and notably, low expression of PD-L1 [238, 239, 275–277]. Overexpression of LKB1 in KRAS mutant NSCLC mice model led to PD-L1 upregulation [278]. Like the interesting result in TP53 co-mutation, even ICI treatment in LKB1-deficient LUAD patients with high PD-L1 expression remains poor performance [238], potentially due to an extensively suppressive immune microenvironment. A real-world observational study showed that KRAS/LKB1 mutation was a negative prognostic factor in LUAD, irrespective of ICI use. LKB1 mutation could not work as the predictive factor influencing the selection of therapy for patients [279], so another explanation is that the poor treatment outcome is due to the overall worse prognosis caused by the other mutated genes and not related to a particular treatment. Despite this explanation, immunosuppression is objective, and in addition to the mechanisms described above, the suppression of STING pathway [280] and activation of inflammatory STAT3 pathway with upregulation of inflammatory factors G-CSF, IL-1α, IL-6, etc [275]. were also involved in the formation of immunosuppression.

Expectations for significant immunotherapy benefits in KRAS/LKB1 co-mutated cancer patients are tempered, yet LKB1 deficiency may enhance responsiveness to other treatment modalities. Mukhopadhyay et al. [281] discovered synthetic lethal genes in KRAS/LKB1 co-mutated NSCLC that bolster efficacy of KRAS^G12C^ inhibitors, implicating components of autophagy, Hippo and c-Myc pathways, thus opening avenues for novel therapeutic strategies. Furthermore, evidence suggested that autophagy inhibition sensitized KRAS/LKB1 co-mutated NSCLC to MEK inhibition, thereby promoting ferroptosis [282]. Therefore, the combined inhibition of autophagy and KRAS emerges as a promising therapeutic strategy. A Phase III clinical trial observed improved OS with the combination of atezolizumab, bevacizumab, and carboplatin/paclitaxel chemotherapy, surpassing the efficacy of each monoclonal antibody combined with chemotherapy alone [283]. Similarly, KRAS/LKB1 co-mutated cells with wild-type TP53 benefited from the combination of trametinib and chemoradiation, which showed resistance when administered separately [284]. Combined cisplatin with metformin [285], combined inhibition of SRC, PI3K, and MEK1/2 [286], HSP90 inhibition [239], FAK inhibition [287], hexosamine biosynthesis pathway inhibition [288], and a mitochondrial inhibitor phenformin [289] have been found effective in KRAS/LKB1 co-mutated lung cancer models. For immunotherapy, despite current obstacles, research into converting “cold” tumors into “hot” ones may herald future breakthroughs [290]. Recent studies have shown that in KRAS-mutant lung cancer cells, YAP1 was the major driver of global RNA expression profile changes caused by LKB1 loss [291] revealing a potential target. Meraz and colleagues [292] utilized nanovesicles to deliver tumor suppressor gene TUSC2 and found improvement in the efficacy of carboplatin plus pembrolizumab in KRAS/TP53 co-mutated NSCLC, mediated by immune microenvironment regulation. Qiao et al. [293] demonstrated that targeting FAK effectively solved the physical barrier for immune cells in KRAS/LKB1 co-mutated cancer, boosting the immune response in the microenvironment. Best et al. [272] found elevated glutamate levels, crucial for T cell activation, accompany KRAS/LKB1 co-mutated LUAD, alongside increased glutamine uptake and glutaminase activity in tumor cells. They reflected a possible benefit from glutaminase monotherapy for KRAS/LKB1 treatment, and combining this with KRAS inhibitors and/or proper immunotherapy is a promising field. For example, LKB1 reconstitution combined with DNMT1/EZH2 inhibition shifted the immune microenvironment significantly [280].

### KRAS/KEAP1 co-mutation

KEAP1 is the regulator upstream of the well-known oxidative and electrophilic stress defender NRF2, and the KEAP1-NRF2 pathway works as the oncogenic signaling to provide survival advantage to cancer cells after activation [294]. For KEAP1 can repress NRF2 by binding to it, the mutation usually leads to activation of that oncogenic signaling [295]. Integrative multi-omics data analysis of KRAS-mutated LUAD patient-derived samples and cell lines showed a significant difference in KEAP1 mutation status between clusters [240]. Although the detailed mechanism is waiting to be elucidated, as a significant character of a subgroup of KRAS-mutated cancer cells, KEAP1 could have a crucial role in shaping global characteristics, or at least as a substantial component. Like KRAS/LKB1 co-mutation, KRAS/KEAP1 co-mutation is a negative prognostic factor in KRAS mutant cancer. It was identified as the independent prognostic factor for survival, platinum-based chemotherapy response, immune therapy outcome [236, 296, 297] and even the negative independent determinant prognostic factor of KRAS^G12C^ inhibitor monotherapy, demonstrated by meta-analysis [298]. In a nationwide cohort study in the USA involving 2593 NSCLC patients, it was found that the KRAS/KEAP1 co-mutation was also associated with low expression of PD-L1 and poor prognosis after ICIs treatment, and triple mutation of KRAS/KEAP1/LKB1 exhibited the poorest outcome [299]. Lower CD8^+^ T cell, B cell, and higher mesenchymal stem cell infiltration were observed in KRAS/KEAP1 co-mutated LUAD patient data [247], reflecting the “cold” immune microenvironment.

Research on the mechanism and therapeutic solution in KRAS/KEAP1 mutant cancer is limited, maybe because of the relatively low mutant rate. Sun et al. [300] reported that NRF2 activation led to the suppressive immune microenvironment through STING inhibition. The secretion of CCL5 and CXCL10 was decreased to reduce the migration of CD8^+^ T cells in vivo with KRAS/KEAP1 co-mutated NSCLC cells. These findings offer insights into the idea of converting “cold” tumors to “hot” in KRAS/KEAP1 co-mutant cancer, and further basic research is needed to find more appropriate therapeutic options.

### Other co-mutations with KRAS

The three kinds mentioned above of co-mutation are most prevalent in KRAS mutant cancer, especially NSCLC. There are other mutations that have relatively lower mutant rates but also have diagnostic, prognostic, or therapeutic influence in KRAS mutant cancer. However, research is limited and presents contradictions among various studies.

KRAS/SMARCA4 co-mutation was identified to confer a poor outcome to immunotherapy in NSCLC [301], with multi-cohort analyses of LUAD yielding similar findings [302, 303]. The research found improvement in OS of SMARCA4 mutant patients who received ICIs compared with those who did not [303], and the authors attributed it to ICI benefits. There was no distinct resistance to ICI in most SMARCA4 mutant patients, even though SMARCA4 mutation status was not enough to be a predictive factor for ICI selection because it was found there was no significant difference in OS or PFS between different SMARCA4 mutation statuses [303].

Computational biological modeling identified KRAS/TP53 and KRAS/PIK3CA co-mutations as biomarkers of anti-PD-(L)1 immunotherapy sensitivity [304]. ATM mutation associated with KRAS mutation in NSCLC has the potential to predict ICI sensitivity because of STING signaling upregulation when treated with chemotherapy [305]. Besides, data from a small cohort of 87 NSCLC participants suggested that the prognosis for immunotherapy in patients with KRAS mutations was significantly worse when co-mutated with CDKN2A/B [137].

Although these co-mutations do not account for as much as the three top co-mutations mentioned above, clarifying their mechanisms is of importance for clinical treatment selection, and the co-mutation and typing of KRAS are bound to evolve rapidly.

### Summary of co-mutation

As a result of the above discussion, the importance of co-mutations in diagnosing and treating tumors with KRAS mutations is illustrated. Much work is still waiting to be done in related research, and the study can be more detailed and in-depth, especially in subgrouping. For example, Aredo et al. [306] pinpointed the KRAS mutation to a distinct type, noting that it was the KRAS^G12D^/LKB1 co-mutation that led to a poorer prognosis and KRAS^G12C^/TP53 co-mutation led to a better prognosis for immunotherapy, which was consistent with the PD-L1 expression pattern reviewed above. The study performed by Cao et al. also provided a good example. They found that LKB1 mutations had no significant impact on PD-L1 expression, but the co-occurrence of KRAS^G12D^ mutations with LKB1 mutations was associated with poorer immunotherapy efficacy [111]. As the role of KRAS mutation type in co-mutation is still unclear and detailed classification could better guide therapy development, similar analysis is necessary.

From an immunotherapy perspective, tumors with KRAS/LKB1 or KRAS/KEAP1 co-mutations are considered as “immune-cold” tumors. Through comprehensive studies of the immune microenvironment in these tumors, we can clarify the mechanisms of immune inhibition in this type of cancer, and specifically use precision immune modulation therapies to reprogram these tumors from an immune “cold” to an immune “hot” state [307]. Alternatively, these co-mutations could serve as biomarkers for treatment selection, avoiding current ICI therapies and developing more effective treatments that align with their underlying mechanism. For example, LKB1-deficient NSCLC cells activated JNK signaling, increasing MCL-1 dependency. Combining MCL-1 inhibitors (e.g., AMG 176) with KRAS/MEK inhibitors showed efficacy and safety in PDX models [308]. Whether this treatment strategy can improve the immune microenvironment, and whether the corresponding inhibitors can be combined with immunotherapy for enhanced efficacy remains an important area for further investigation. Additionally, NRF2 and glutaminolysis dependency downstream of KEAP1 mutations are promising therapeutic targets [309]. Both approaches require further basic research into the immune microenvironment and tumor biology of such co-mutation tumors, particularly in NSCLC.

We refer to co-mutation as a potential challenge because its presence complicates the problem. However, from an immunotherapeutic perspective, classification based on KRAS mutation type seems to lack a high degree of guidance, and the above summary reveals that there are too many exceptions that make the pattern difficult to summarize. In this context, the immune microenvironmental alterations that characterize co-mutations appear clearer, so this potential challenge is likewise a potential chance to solve the immunotherapy problem. We look forward to improving the co-mutation-based tumor typing paradigm for KRAS mutations in the future, intending to guide clinical immunotherapy.

## Future perspective

This review comprehensively summarized the distinctions in the immune microenvironment among different types of KRAS mutations, particularly KRAS^G12D^ and KRAS^G12C^, and emphasized the critical role of various co-mutations in shaping the heterogeneity of the KRAS tumor immune microenvironment. By reviewing the history and current status of KRAS-targeted therapies, it is easy to recognize that while significant strides have been made, issues such as resistance, toxicity, and limited efficacy indicate that these are incremental steps in the battle against KRAS. KRAS mutations exert a comprehensive and potent regulatory effect on immune microenvironment reprogramming, involving a broad spectrum of immune-related pathways and various immune cell types in the tumor milieu. It underscores the significant role of the immune microenvironment in the development and progression of KRAS-mutant tumors. It suggests that modulating this environment could be vital to breaking the impasse in treating these tumors.

Research into the immune microenvironment of KRAS-mutant tumors faces several challenges. Most importantly, precise subtyping of KRAS mutations and co-mutations is essential, as these mutations are the primary drivers of heterogeneity in the KRAS mutant tumor immune microenvironment—and this extends beyond just the immune landscape. Studies that do not differentiate subtypes of KRAS mutations are increasingly seen as insufficient for providing high clinical value, and it is hoped that future research will address this. Co-mutations present challenges for precise subtyping of KRAS mutations but also offer chances for resolution. The ways of crosstalk between KRAS and other mutations is still unclear. Further in-depth study on the molecular mechanisms of co-mutation-induced changes in the immune microenvironment and other areas is essential for a comprehensive understanding of KRAS-mutant tumors.

Additionally, research into the KRAS mutation immune microenvironment is not yet comprehensive, whether in terms of basic mechanisms or the analysis of real-world clinical data. The ideal development is to achieve precise regulation of components within the immune microenvironment, reversing immune reprogramming to treat KRAS mutant tumors, making a comprehensive understanding of the KRAS mutation immune microenvironment crucial for precision immune regulatory therapy. Moreover, for oncogenes like KRAS, the conclusions drawn from mechanistic studies are likely to differ from real-world outcomes. In basic research, KRAS-mutant tumor models typically use KRAS mutations as the oncogenic driver. In reality, the presence of a KRAS mutation does not necessarily imply it is a driver gene; KRAS mutations may be acquired during the tumor evolution process. Therefore, the role of KRAS mutations may also vary, which could be a significant reason for the discrepancies between basic research on these oncogenes and clinical outcomes. On one hand, experimental designs can investigate the impact of such differences, while on the other hand, real-world clinical studies are crucial. Hence, a more detailed and extensive study of the immune microenvironment is necessary.

Lastly, the exploration of existing drug combinations remains crucial. Since targeting the ideal immune microenvironment requires the development of corresponding drugs, we are still far from implementing precise targeting of the immune microenvironment in clinical settings. Exploring combinations of KRAS inhibitors and existing immunotherapies holds direct practical value, whether exploring new drug combinations or further researching the toxicity and efficacy mechanisms of KRAS^G12C^ inhibitors in combination with ICIs.

Through this review, we have recognized the unique role of KRAS mutations as a critical oncogene in shaping the immune microenvironment. We believe this represents a promising direction for both the study of KRAS mutation-driven carcinogenesis and the future development of precision medicine for KRAS-mutant cancer patients. Currently, research into the immune microenvironment characteristics of KRAS-mutant tumors remains insufficiently detailed, while the available immunotherapy options are limited, with most established treatments focusing on immune checkpoint inhibitors. By further refining the understanding of immune microenvironment features and regulatory mechanisms across various KRAS mutation subtypes (including different co-mutations) through preclinical studies and even clinical trials, along with the continuous development of immunotherapy approaches, it will be only a matter of time before precise immune modulation for KRAS-mutant tumors enters clinical treatment guidelines.

## Data Availability

No datasets were generated or analysed during the current study.

## Abbreviations

AICD: Activation-induced cell death
CRC: Colorectal cancer
CYPA: Capable of remodeling cyclophilin A
DC: Dendritic cell
ICI: Immune checkpoint inhibition
LUAD: Lung adenocarcinoma
MDSC: Myeloid-derived suppressor cell
MHC: Major histocompatibility complex
NET: Neutrophil extracellular trap
NO: Nitric oxide
NSCLC: Non-small-cell lung cancer
PFS: Progression-free survival
OS: Overall survival
TAM: Tumor-associated macrophages
TMB: Tumor mutational burden
